# Self-supervised learning of molecular representations from millions of tandem mass spectra using DreaMS

**DOI:** 10.1038/s41587-025-02663-3

**Published:** 2025-05-23

**Authors:** Roman Bushuiev, Anton Bushuiev, Raman Samusevich, Corinna Brungs, Josef Sivic, Tomáš Pluskal

**Affiliations:** 1 Institute of Organic Chemistry and Biochemistry of the Czech Academy of Sciences, Prague, Czech Republic; 2 Czech Institute of Informatics, Robotics and Cybernetics, Czech Technical University, Prague, Czech Republic

**Keywords:** Machine learning, Metabolomics, Mass spectrometry

## Abstract

Characterizing biological and environmental samples at a molecular level primarily uses tandem mass spectroscopy (MS/MS), yet the interpretation of tandem mass spectra from untargeted metabolomics experiments remains a challenge. Existing computational methods for predictions from mass spectra rely on limited spectral libraries and on hard-coded human expertise. Here we introduce a transformer-based neural network pre-trained in a self-supervised way on millions of unannotated tandem mass spectra from our GNPS Experimental Mass Spectra (GeMS) dataset mined from the MassIVE GNPS repository. We show that pre-training our model to predict masked spectral peaks and chromatographic retention orders leads to the emergence of rich representations of molecular structures, which we named Deep Representations Empowering the Annotation of Mass Spectra (DreaMS). Further fine-tuning the neural network yields state-of-the-art performance across a variety of tasks. We make our new dataset and model available to the community and release the DreaMS Atlas—a molecular network of 201 million MS/MS spectra constructed using DreaMS annotations.

## Abstract (web summary)

A transformer model is used to construct the DreaMS Atlas—a molecular network of 201 million MS/MS spectra.

## Main

The discovery and identification of small molecules and metabolites impacts various scientific fields, including drug development^1^, environmental analysis^2^ and disease diagnosis^3^. However, only a tiny fraction of natural small molecules have been discovered to date, estimated to be less than 10% of those present in the human body or the entire plant kingdom^4^. Most of the natural chemical space remains unexplored.

Tandem mass spectrometry coupled with liquid chromatography (LC–MS/MS) is an analytical technique for investigating the molecular composition of biological and environmental samples. When analyzing a sample, the LC–MS/MS system separates molecules through liquid chromatography, ionizes them and records their mass-to-charge ratios (*m*/*z*), generating a series of mass spectra (referred to as MS^1^). Each MS^1^ spectrum is acquired at a specific retention time and represents the abundance of ions in terms of their *m*/*z* ratios (that is, peaks). Using a technique referred to as data-dependent acquisition, selected ions (referred to as precursor ions) undergo fragmentation, typically using collision-induced dissociation (CID), yielding additional tandem mass spectra (referred to as MS^2^ or MS/MS), where signals characterize molecular fragments of a single selected ion. Although MS^2^ and deeper MS^*n*^ tandem mass spectra constitute the primary source of structural information in mass spectrometry, their interpretation remains challenging. In particular, a mere 2% of MS/MS spectra in an untargeted metabolomics experiment can be annotated with molecular structures using reference spectral libraries^5,6^, and less than 10% of MS/MS spectra can typically be annotated using state-of-the-art machine learning tools^7^.

Existing methods for the interpretation of mass spectra of small molecules can be classified into three major categories: spectral similarity, forward annotation and inverse annotation. Spectral similarity algorithms aim to define a similarity measure on mass spectra, which reflects the similarity of the underlying molecular structures. Classic dot-product-based algorithms are optimized for querying spectral libraries and linking spectra of similar compounds into molecular networks^8–10^. Unsupervised shallow machine learning methods, MS2LDA^11^ and Spec2Vec^12^, devise more versatile spectral similarities based on statistical occurrences of spectral peaks. By contrast, recently developed contrastive learning approaches explicitly approximate similarities in molecular structures^13–15^. The use of similarity-based methods is heavily dependent on the richness of annotated spectral libraries, which are inherently limited in size^16^. Therefore, forward annotation methods extend MS/MS datasets with in silico spectra by simulating CID fragmentation of molecules via combinatorial optimization based on hand-crafted priors^17,18^ or graph neural networks^19–21^. Contrastingly, inverse annotation methods directly annotate spectra with molecular structures, in the approximate form of molecular fingerprints^22^, molecular formulas^23,24^, chemical properties^25,26^ or as complete de novo molecular structures^27–29^.

**Fig. 1:**
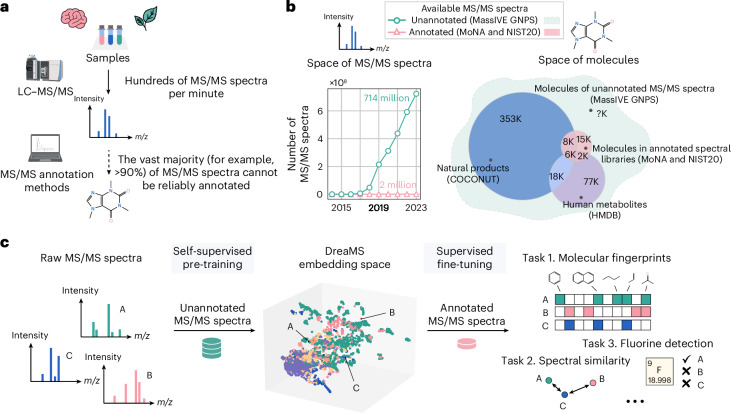
The DreaMS neural network overcomes the limitation of mass spectral libraries. **a**, Given a biological or environmental sample, the LC–MS/MS system produces hundreds of mass spectra (MS/MS) per minute, characterizing its molecular composition. However, less than 10% of MS/MS spectra of small molecules can typically be assigned with molecular structures using existing annotation methods. **b**, Even though the number of publicly available unannotated experimental mass spectra has been rapidly growing over recent years (left; green), annotated spectral libraries are still highly limited in terms of both the number of spectra (left; pink) and the coverage of molecular structures (right; Venn diagram). State-of-the-art annotation methods rely on spectral libraries as training or retrieval datasets. By contrast, we base our method on training from vast unannotated MS/MS datasets, assuming that the molecular coverage of these data surpasses spectral libraries (right; dashed green shape). K, thousand. **c**, We propose the DreaMS neural network, which is capable of learning molecular representations from raw unannotated mass spectra through self-supervised learning. After being pre-trained in a self-supervised way, DreaMS can be fine-tuned for a wide range of spectrum annotation problems via supervised transfer learning, leveraging spectral libraries as well as other sources of annotated data. The icons were created with BioRender.com.

The most prominent and well-established method for the interpretation of small-molecule mass spectra, SIRIUS^30^, comprises a pipeline of approximate inverse annotation tools based on combinatorics, discrete optimization and machine learning leveraging mass spectrometry domain expertise. First, it explains a given MS/MS spectrum with a fragmentation tree by assigning chemical formulas to individual spectral peaks^24^. Then, it employs a series of support vector machines (SVMs) with kernels, designed to operate on mass spectra and fragmentation trees. SIRIUS predicts a proprietary CSI:FingerID fingerprint^22^, which is used to retrieve a molecular structure from a compound database such as PubChem^31^. Recently developed competitive methods, MIST^32^ and MIST-CF^33^, replace crucial components of SIRIUS with neural networks trained on spectral libraries. Both methods employ a similar transformer architecture that operates on chemical formulas assigned to individual peaks as input tokens. Whereas MIST-CF assigns chemical formulas through energy-based modeling, MIST uses these formulas to predict a molecular fingerprint and employs it to retrieve a molecular structure from compound databases. To achieve a level of performance that is competitive with SIRIUS, both methods employ additional domain-specific computationally demanding components, such as mass decomposition^34^, data pseudo-annotation with the forward annotator MAGMA^35^ or the generation of in silico spectral libraries^32^. The reliance of the state-of-the-art machine learning models on a variety of auxiliary methods suggests that the capacity of training spectral libraries is the principal bottleneck of the process. In fact, the molecular structures of the standard training spectral libraries MoNA^36^ and NIST20 (ref. ^37^) cover only a limited subset of known natural molecules (Fig. 1b), not to mention the vastness of the chemical space that remains to be explored.

Here we introduce a large self-supervised neural network (with 116 million parameters) trained directly on the repository-scale collection of raw experimental mass spectra of small molecules (Fig. 1b,c). Inspired by the achievements of large transformer models pre-trained in a self-supervised way on biological protein sequences^38–42^, text^43,44^ and images^45^, we developed a transformer model for MS/MS named Deep Representations Empowering the Annotation of Mass Spectra (DreaMS). Without relying on prior methodologies or human domain expertise, DreaMS can be adapted to a wide range of spectrum annotation tasks and act as a foundation model for MS/MS^46^. To achieve this, we first constructed a high-quality dataset, GNPS Experimental Mass Spectra (GeMS), comprising up to 700 million MS/MS spectra mined from the Global Natural Products Social Molecular Networking (GNPS) repository^47^. Second, we designed a transformer neural network and pre-trained it on our GeMS data to predict masked spectral peaks and chromatographic retention orders (the ‘Related work in supervised learning on mass spectra’ subsection in Methods discusses methodological differences with related supervised methods). We show that, through optimization toward these self-supervised objectives on unannotated mass spectra, our model discovers rich representations of molecular structures. Specifically, we found that the DreaMS representations (1,024-dimensional real-valued vectors) are organized according to the structural similarity between molecules and are robust to mass spectrometry conditions. We demonstrate that DreaMS, fine-tuned for diverse mass spectrum annotation tasks, including the prediction of spectral similarity, molecular fingerprints, chemical properties and the presence of fluorine, surpasses both traditional algorithms and recently developed machine learning models. Finally, we applied the fine-tuned models to construct DreaMS Atlas—a molecular network of 201 million MS/MS spectra assembled using DreaMS annotations.

## Results

### Large-scale datasets of MS/MS spectra for deep learning

Comprehensive and high-quality datasets are essential for effective self-supervised learning^48–50^. However, spectral libraries of metabolites are limited in size and cover only a tiny fraction of the entire chemical space. To our knowledge, there are no large standardized datasets of mass spectra suitable for unsupervised or self-supervised deep learning. Therefore, we mined the MassIVE GNPS repository^47^ to establish a new large-scale and high-quality dataset comprising hundreds of millions of experimental MS/MS spectra, which we named GeMS.

**Fig. 2:**
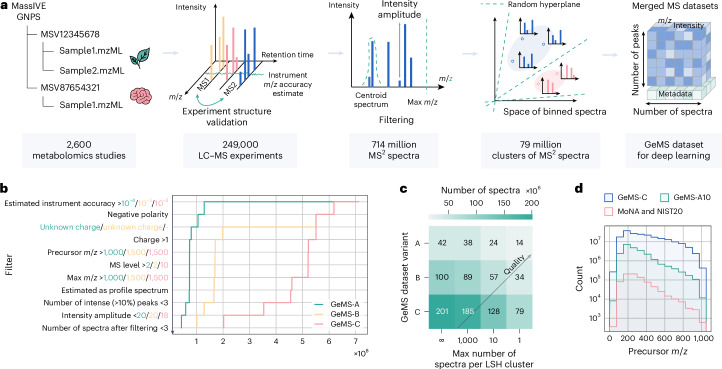
GeMS—high-quality datasets of unannotated MS/MS spectra from GNPS. **a**, The workflow of mining GeMS datasets from the GNPS repository. MS/MS spectra from metabolomics studies were filtered using experiment-level and spectrum-level quality criteria, clustered with LSH and packed into a tensor-like dataset suitable for deep learning. **b**, Quality criteria defining the A, B and C subsets of GeMS data, listed in order of their application from top to bottom (Supplementary Table 1). **c**, Sizes of the three final unclustered (first column) and nine clustered (last three columns) GeMS variants. Each cell in the heatmap corresponds to a specific variant, denoted in the text as, for instance, GeMS-A10, based on the respective axes. Seventy-nine million clusters on top represent the fully clustered GeMS-C1 subset of GeMS-C. **d**, All the GeMS dataset variants are orders of magnitude larger than the union of MoNA and NIST20 spectral libraries and cover a wide range of molecular masses.

**Extended Data Fig. 1:**
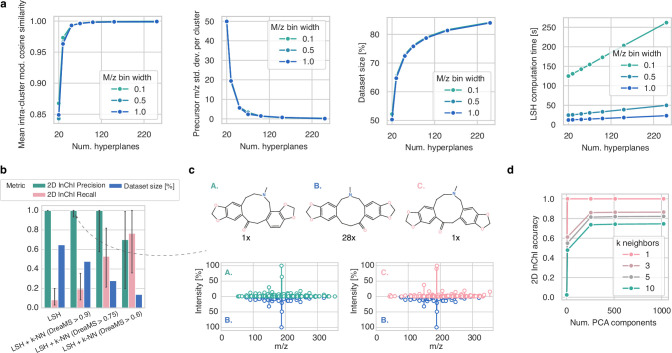
Benchmarking of MS/MS clustering with LSH and DreaMS embeddings. **a**, The figure evaluates locality-sensitive hashing (LSH) clustering of MS/MS spectra (n = 94,837 spectra) under varying m/z bin widths and numbers of hyperplanes. As shown, increasing the number of hyperplanes improves LSH clusters in approximating cosine similarity and preserving precursor m/z values, while more spectra remain unclustered. Notably, the m/z bin width has a minor effect on the cluster quality but substantially influences the algorithm’s running time. **b**, The leftmost column demonstrates the performance of LSH clustering (30 hyperplanes and bin width of 1.0) in grouping spectra from identical compounds. The bars show median values, while error bars represent the corresponding interquartile ranges (n = 94,837 spectra). The remaining bars illustrate the combination of LSH clusters into larger clusters to construct the DreaMS Atlas. The grouping is based on the breadth-first search (BFS) aggregations in the k-NN graph of the DreaMS Atlas under different DreaMS similarity cutoffs. A cutoff of 0.9 enhances the recall of LSH clustering while maintaining a median precision of 1 (second group of bars). Lower cutoffs further increase recall but also introduce more false positives. To ensure that most of the nodes in the DreaMS Atlas represent single molecular structures, we used the 0.9 cutoff for its construction. **c**, The figure provides an example of a cluster refined using DreaMS k-NN with a 0.9 cutoff, resulting in a precision slightly below 1.0. The cluster contains 30 spectra, of which 28 correspond to the same structure, while two are different but highly similar. Despite the variation, all structures are closely related and exhibit highly similar, potentially indistinguishable, MS/MS spectra. **d**, The figure generalizes Fig. 3e, quantitatively showcasing the linear organization of self-supervised DreaMS embeddings in the affine PCA subspace with respect to molecular structures, thus demonstrating their robustness to mass spectrometry conditions. In the 1,023-dimensional subspace (embedding dimensionality minus one), the accuracy reaches 0.865 for k = 3 and 0.999 for k = 1, highlighting the linearity of the embedding space in clustering spectra from identical compounds. Additional details can be found in the Online Methods.

Our mining pipeline consists of five main steps (Fig. 2a). First, we collected 250,000 LC–MS/MS experiments from diverse biological and environmental studies, covering virtually the entire GNPS part of the MassIVE repository^47^. Second, we extracted from these experiments approximately 700 million MS/MS spectra. Next, we developed a pipeline of quality control algorithms allowing us to filter the collected spectra into three subsets—GeMS-A, GeMS-B and GeMS-C—each with consecutively larger size at the expense of quality. The quality criteria include, for example, the estimation of the instrument *m*/*z* accuracy associated with a single LC–MS/MS experiment or the number of high-intensity signals within each spectrum (Fig. 2b). For reference, 97% of spectra in the highest-quality GeMS-A subset were acquired using Orbitrap mass spectrometers, whereas the GeMS-C subset comprises 52% Orbitrap and 41% quadrupole time of flight (QTOF) spectra. Subsequently, we addressed redundancy in GeMS by clustering similar spectra using locality-sensitive hashing (LSH). The LSH algorithm approximates cosine similarity (Extended Data Fig. 1a), a common metric for identifying similar spectra, but operates in linear time, enabling efficient clustering of our large-scale data. Specifically, we limited cluster sizes to a certain number of randomly sampled spectra per cluster, such as 10 or 1,000, resulting in a total of nine different GeMS dataset variants (Fig. 2c). Finally, we stored the GeMS spectra, including selected LC–MS/MS metadata, in our compact HDF5-based binary format designed for deep learning (Supplementary Table 2). Our new GeMS datasets are orders of magnitude larger (Fig. 2d) than existing spectral libraries and are well organized into numeric tensors of fixed dimensionality, unlocking new possibilities for repository-scale metabolomics research^51,52^. The details of the data collection and filtering are provided in Methods.

### Self-supervised pre-training on MS/MS spectra

Leveraging the GeMS-A10 dataset, our highest-quality subset of GeMS, we propose DreaMS—a self-supervised model that learns molecular representations directly from unannotated mass spectra. Self-supervision is a form of unsupervised learning, where the training objective typically involves a reconstruction of corrupted data points. This approach has been demonstrated to yield rich representations (that is, embeddings) of words, images or proteins, which effectively generalize across diverse tasks^39,43,45^. Although self-supervised learning has been applied in the contexts of mass spectrometry imaging^53^, proteomics^54^ and liquid chromatography^55,56^, it has not yet been explored for MS/MS spectra of small molecules. This is primarily due to the lack of large, standardized datasets and strong inductive biases necessary for large-scale learning. We addressed this challenge by designing a transformer-based neural network tailored for MS/MS spectra and training it using our new large-scale dataset.

**Fig. 3:**
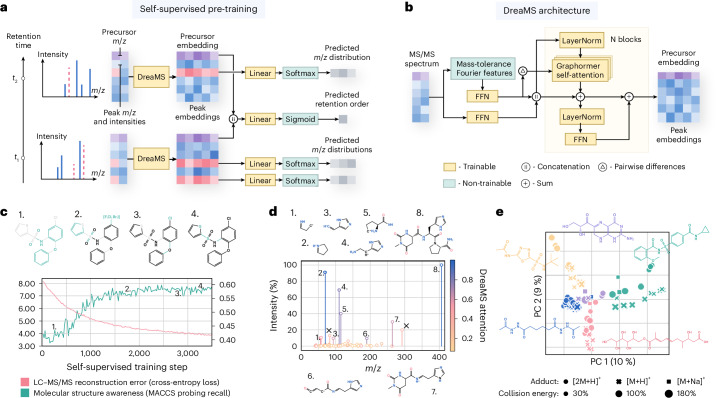
The DreaMS neural network discovers molecular structures through self-supervised learning on mass spectra. **a**, Self-supervision setup. The DreaMS neural network is provided with a pair of spectra (blue) from the same LC–MS/MS experiment along with their precursor *m*/*z* values (purple). A portion of *m*/*z* ratios in both spectra is masked (red), and the model is trained to reconstruct these values by predicting a probability distribution over *m*/*z* ratios for each mask. Additionally, the model learns to predict the retention order of the two spectra (that is, the probability that *t*_2_ > *t*_1_). **b**, Architecture of the DreaMS neural network. Initially, input spectral peaks, including precursor *m*/*z* with artificial intensity value, are assigned mass-tolerance Fourier features and processed with shallow feed-forward neural networks (FFNs). The subsequent transformer encoder, equipped with Graphormer self-attention layers operating on pair-wise mass differences, refines the encoded peaks into high-dimensional output embeddings. **c**, Emergence of molecular structures from self-supervised training. At each self-supervised training step, DreaMS parameters are frozen, and a separate linear layer is trained to predict interpretable MACCS keys fingerprints from precursor peak embeddings, allowing the inspection of learned molecular fragments. As the self-supervised loss decreases (red), the recall in MACCS bits increases (green), indicating the model’s ongoing discovery of new molecular structures. The MACCS fragments for individual bits are visually presented on top. **d**, An example spectrum colored based on the maximum attention value across all attention heads for each peak (blue indicates high attention, yellow indicates low attention). DreaMS focuses on high-intensity peaks that appear to represent molecular fragments and ignores noise. Molecules depict fragment annotations produced by Mass Frontier (Thermo Fisher Scientific); crossed intense peaks lack annotations. **e**, PCA applied to selected precursor embeddings demonstrates the linear clustering of mass spectra according to molecular structures, remaining robust to multiple ionization adducts and normalized collision energies associated with each molecule. PC, principal component.

The core of our self-supervised approach (Fig. 3a) is BERT-style^43^ spectrum-to-spectrum masked modeling. We represent each spectrum as a set of two-dimensional (2D) continuous tokens associated with pairs of peak *m*/*z* and intensity values. Then, we mask a fraction (30%) of random *m*/*z* ratios from each set (or spectrum), sampled proportionally to corresponding intensities, and train the model to reconstruct each masked peak. Additionally, we introduce an extra token, which we refer to as the precursor token. This token is never masked and contains a precursor ion *m*/*z* ratio and a precursor-specific artificial intensity value, serving as an aggregator of spectrum-level information into a single embedding, akin to a sentence-level token or a graph-level master node in the related language of graph models^43,57^. Besides masked *m*/*z* prediction, we employ a retention order training objective. Each training example is formed as a pair of partially masked spectra, sampled from the same LC–MS/MS experiment, and the neural network simultaneously learns to reconstruct the masked peaks and to predict which one elutes first in chromatography.

The backbone of our DreaMS neural network architecture (Fig. 3b) is based on the transformer encoder^58^ (seven layers). It consists of a sequence of multi-head self-attention blocks (8 heads in each block), which gradually derive the representations of peaks (1,024-dimensional real-valued vectors) and relationships between them. We adjust the standard architecture to handle high-resolution molecular masses. First, each *m*/*z* value is pre-processed with a modification of Fourier features, a computer vision technique shown to improve the representation of high-resolution details in images^59^. In essence, each *m*/*z* value is decomposed into pre-defined sine and cosine frequencies capturing both the integer and the floating-point part of a single mass. We additionally process the Fourier features with a feed-forward network to enable, for example, the learning of possible elemental compositions associated with input masses^60^. We incorporate intensity values by processing them through a shallow feed-forward network and then concatenating them with the processed Fourier features. This combined representation serves as the input for the transformer. Second, we explicitly feed differences in Fourier features between all pairs of peaks to self-attention heads, following the Graphormer architecture^61^. This enables the transformer to attend directly to neutral losses without increasing computational complexity through the introduction of extra tokens or modifications to the dot-product attention mechanism. Finally, instead of treating masked *m*/*z* prediction as a regression problem, we treat it as classification and train the model to predict a probability distribution over a binned mass range for each mask. This approach allows the network to model the uncertainty of predictions when multiple *m*/*z* values could match the same intensity.

**Extended Data Fig. 2:**
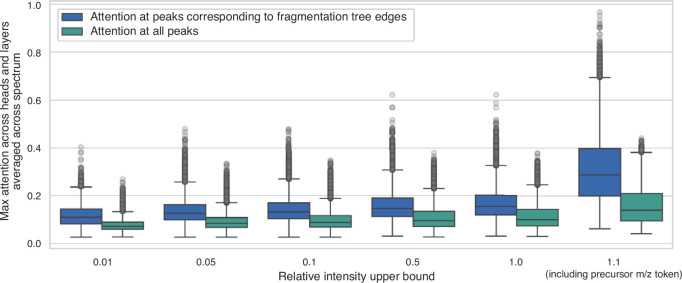
The pre-trained DreaMS model prioritizes attention to peaks that can be explained by SIRIUS fragmentation trees. The evaluation was conducted as follows: First, we performed a forward pass through the DreaMS model for random MS/MS spectra sampled from the MassSpecGym dataset^98^ (n = 100,000 spectra). For each spectrum, we extracted attention score matrices across all attention heads and layers, resulting in tensors of shape (num_layers, num_heads, num_peaks, num_peaks). Second, we applied maximum pooling across the number of layers and the number of heads, yielding square matrices of shape (num_peaks, num_peaks), which represent the maximum attention paid to each pair of peaks across the entire transformer model. The green box plots in the figure show the distribution of mean values in these matrices when selecting elements representing the attention scores between peaks with relative intensity not exceeding a certain value (x-axis). The intensity upper bound of 1.1 denotes all peaks, the intensity upper bound of 1.0 denotes all peaks except for the precursor token peak, and so on. Third, we annotated all spectra using SIRIUS fragmentation trees and plotted the attention means when retaining only pairs of peaks that are present in the trees as edges (blue box plots). The figure demonstrates that the attention paid to pairs of peaks, which are explained by fragmentation trees, is significantly higher than the overall attention under different intensity cutoffs (Mann-Whitney U test p-value < 0.05 for each upper bound). In this evaluation, we considered only spectra with more than ten peaks, more than five pairs of peaks after applying the intensity cutoff, and at least 50% of peaks explained by the SIRIUS fragmentation trees. Each box plot displays the median (center line), quartiles (box bounds), whiskers extending to 1.5 times the interquartile range, and outliers beyond.

We hypothesize that when the DreaMS model is trained to predict masked *m*/*z* ratios and chromatographic retention orders, it implicitly learns to reason in terms of molecular structures. To test this hypothesis empirically, we first employed a machine learning technique called linear probing^62^ to assess the evolution of learned representations during training. Specifically, when training a simple logistic regression from precursor embeddings to interpretable MACCS keys fingerprints^63^ at each training step, we noted that, during self-supervised training, the model progressively discovers molecular fragments (Fig. 3c). Second, our analysis of transformer attention heads revealed that the model learned to prioritize peaks that appear to represent molecular structures over the noisy signals (Fig. 3d and Extended Data Fig. 2). Third, we found that the DreaMS representation space linearly clustered spectra according to molecular structures, even when fragmented under different ionization and fragmentation settings (Fig. 3e and Extended Data Fig. 1d).

**Extended Data Fig. 3:**
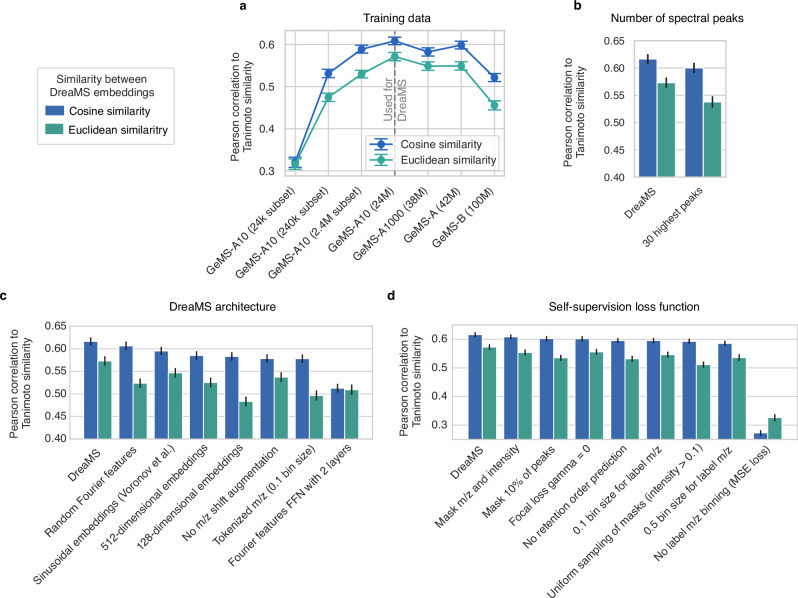
Ablation study of self-supervised pre-training. The evaluated metrics are Pearson correlation coefficients between cosine similarity and Euclidean similarity (the inverse of Euclidean distance) on DreaMS embeddings with Morgan Tanimoto molecular similarity. The bars display maximum values of the metrics achieved through the course of pre-training with each of the model configurations. **a**, Comparison of different dataset configurations (values in brackets show dataset sizes with ‘k’ standing for thousands and ‘M’ standing for millions). The figure demonstrates that (i) the performance of pre-training consistently increases with the amount of high-quality clustered spectra from GeMS-A10 but (ii) deteriorates when including more loosely clustered spectra (GeMS-A1000 and GeMS-A) or lower-quality spectra (GeMS-B). **b**–**d**, Comparison of different DreaMS configurations across three categories: numbers of peaks in each spectrum used for training (**b**), DreaMS architecture components (**c**), and modifications of the self-supervision loss function (**d**). DreaMS represents the final model presented in this work, trained on the GeMS-A10 dataset using the sixty highest peaks, Graphormer-like attention mechanism, Fourier features (5 layers for subsequent feed-forward network), 1024-dimensional embeddings (that is, transformer hidden dimensionality), m/z shift augmentations, masking 30% of m/z values (not masking intensities) sampled proportionally to corresponding intensities, focal loss with gamma equal to 5, retention order prediction, and a 0.05 bin size for m/z labels. Sinusoidal embeddings and tokenized m/z refer to peak representations proposed by Voronov et al.^14,25^. The underlying dataset comprises pairs of spectra from NIST20 sampled to maximize the entropy of the Tanimoto similarity distribution (n = 5,000 pairs). Data are presented as mean values with error bars representing standard deviations within 1,000 bootstrap samples.

Ablation studies of the pre-training dataset suggest that DreaMS performance scales with the amount of high-quality clustered data, such as spectra from the GeMS-A10 dataset (Extended Data Fig. 3a). However, consistently with findings in natural language processing^48–50^, data quality appears to be more critical than data scale. Specifically, DreaMS performance deteriorates when incorporating lower-quality or loosely clustered spectra, such as those from GeMS-B. Regarding the DreaMS architecture, the ablations indicate that key components of our self-supervised approach are mass-tolerant Fourier features, and the masked *m*/*z* objective formulated as a classification task rather than regression (Extended Data Fig. 3b–d).

### Transfer learning to MS/MS spectrum annotation tasks

**Fig. 4:**
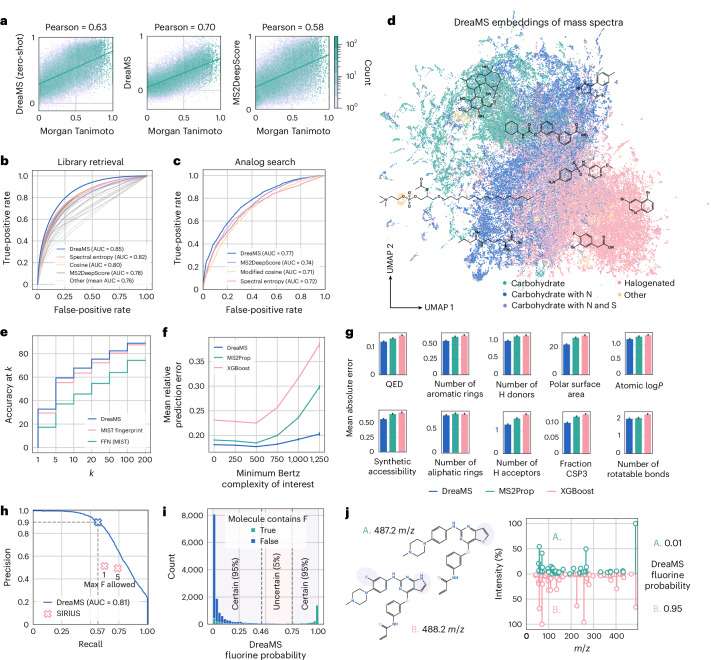
The DreaMS neural network outperforms state-of-the-art methods at solving a variety of spectrum annotation tasks. **a**, Zero-shot (that is, unsupervised) cosine similarity of DreaMS representations outperforms MS2DeepScore^13^ in predicting precursor Tanimoto similarities. Contrastive fine-tuning further enhances the correlation (fine-tuned models are referred to simply as DreaMS; Extended Data Fig. 5a). **b**,**c**, The same fine-tuned model outperforms MS2DeepScore and classic spectral similarity algorithms in the tasks of library retrieval (**b**, that is, retrieval of the same 2D InChI key from a pool of molecules with a 10-ppm *m*/*z* difference) and analog search (**c**, that is, retrieval of molecules with five or fewer structural modifications as measured by the MCES distance^66^; Extended Data Fig. 5b,c). **d**, UMAP projection of DreaMS embeddings reveals the organization of representation space according to molecular formulas. **e**, DreaMS fine-tuned to predict Morgan fingerprints (blue) performs on par with the MIST fingerprint model^32^ (pink), in terms of compound database retrieval accuracy on the MIST benchmark^32^. FFN (MIST) represents a feed-forward neural network baseline proposed in the MIST paper (green). **f**,**g**, DreaMS outperforms existing models in predicting molecular complexity (**f**) and ten other chemical properties^25,26^ (**g**). Error bars show 99% confidence intervals of 1,000 bootstrap samples. Notably, by predicting Bertz complexity, DreaMS excels on practically interesting, high-complexity examples. **h**, DreaMS (blue) surpasses SIRIUS (pink; two different settings) in detecting fluorinated molecules, achieving almost two-fold greater 90% precision under 57% recall on 17,000 test spectra from our new in-house library. **i**, Ninety-five percent of all predictions on the same test spectra can be classified as certain when certainty is defined as falling below the 90% precision threshold for fluorine absence prediction or exceeding the 90% precision threshold for fluorine presence prediction (dashed lines indicate the thresholds). **j**, Model generalization demonstrated on two similar spectra of nearly identical molecules with different fluorine annotations. DreaMS confidently predicts correct annotations despite the absence of similar training examples (Extended Data Fig. 5d). The details on the evaluation datasets and metrics are provided in Methods.

**Extended Data Fig. 4:**
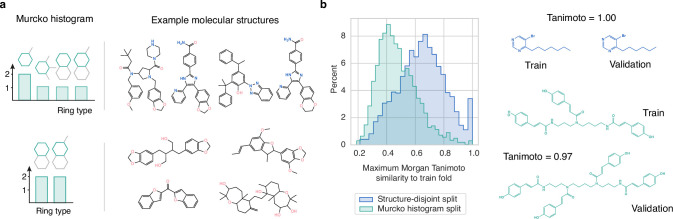
Our Murcko histograms splitting of spectral libraries surpasses the structure-disjoint approach. **a**, Schematic illustration of the Murcko histogram molecular representations. Different molecular structures shown on the right side have identical Murcko histograms shown on the left side. The domain of a Murcko histogram is defined in terms of rings (green structures) of different types based on the number of neighboring rings and linkers (grey structures). The corresponding values are the counts of such ring types. **b**, Evaluation of data leakage on structure-disjoint and Murcko histogram-disjoint splits of MoNA in terms of the maximum Morgan Tanimoto similarity to each training example computed for each validation example. Structure-disjoint splitting results in many leaking near-duplicate examples present in the validation set (maximum Tanimoto similarity > 0.95) whereas Murcko histograms-based splitting eliminates almost all such examples. The blue pair of structures represents such leaks, and the green pair demonstrates that the high-similarity inter-fold examples resulting from Murcko histogram splitting are rather a result of the imperfectness of Tanimoto similarity than the splitting algorithm. The mean of the Murcko histogram-disjoint similarity distribution is approximately one-half the value of the mean of the structure-disjoint-related distribution, implying that our approach to data splitting is better suited for evaluating model generalization.

The emergence of molecular structures in DreaMS is a result of self-supervised training from extensive unannotated mass spectral data, without relying on annotated MS/MS libraries, chemical databases or human expertise. It motivates us to investigate DreaMS as a foundation model possessing a general understanding of molecules, which can be transferred to various spectrum annotation tasks. In particular, we adapted the network to the prediction of spectral similarity, molecular fingerprints, chemical properties and the identification of fluorine-containing molecules. For each task, we augment the pre-trained model with a simple linear head or a shallow feed-forward network and fine-tune the entire neural network end to end on annotated spectral libraries. To ensure the generalization of fine-tuned models beyond spectral libraries, we halt fine-tuning when the model’s performance plateaus on validation spectra of molecules with different Murcko histograms from those in the training set (except for the fingerprint prediction benchmark established by Goldman et al.^32^). A Murcko histogram is our new molecular representation, generalizing the notion of a Murcko scaffold^64^ (described in Methods and Extended Data Fig. 4). This universal transfer learning protocol consistently yields models with state-of-the-art performance across different tasks, eliminating the need for constructing task-specific components or extensively tuning model hyperparameters (Fig. 4).

**Fig. 5:**
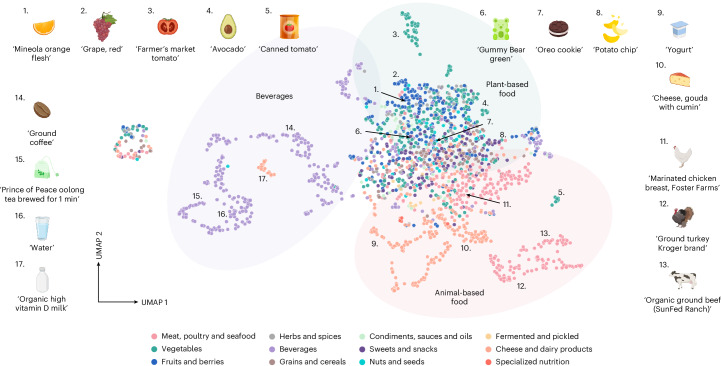
Sample-average DreaMS embeddings enable the sample-level analysis of metabolomics data, as exemplified on food LC–MS/MS datasets. Each point on the UMAP plot represents a centroid of DreaMS embeddings (that is, mean embedding values across dimensions) of all MS/MS spectra acquired from a certain food sample measured on a QTOF instrument^75^. Numbered points indicate selected example samples and refer to their textual descriptions assigned by the data collectors. The figure demonstrates that the space of sample-level embeddings correctly captures the taxonomy of food items presented to DreaMS as collections of MS/MS spectra. Specifically, the space is organized into three major regions predominantly populated with beverages (purple ellipse), plant food items (green ellipse) and animal food items (pink ellipse). Beverages are separated into milk beverages (orange) and other beverages (purple). Animal-based food items are divided into clusters comprising various dairy products (orange) and types of meat (pink). Plant-based food items show less distinction between categories and are primarily classified as vegetables (green), fruits (blue) and herbs and spices (gray). Individual categories (colors) were assigned to sample descriptions using ChatGPT 4 (ref. ^76^). The details are provided in Methods.

**Extended Data Fig. 5:**
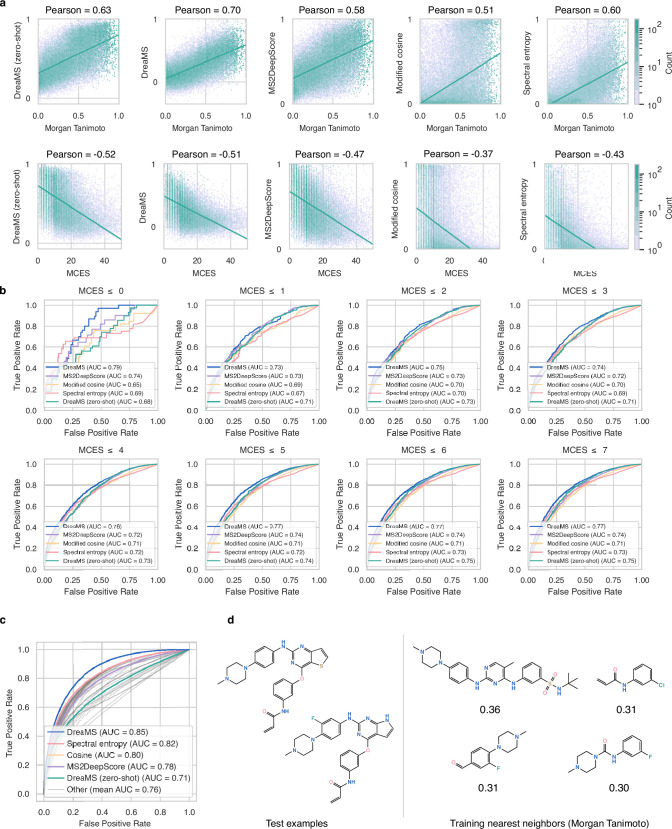
Extended benchmarking results. **a**, Cosine similarity in the space of DreaMS embeddings outperforms the supervised method MS2DeepScore and the classic modified cosine and spectral entropy methods, as measured by two metrics: correlation with Morgan Tanimoto similarity and correlation with the maximum common edge subgraph (MCES) distance. **b**, DreaMS surpasses the other considered methods in an analog search scenario, as measured by the AUROC metric, where positive labels are defined as pairs with MCES distances lower than or equal to a certain threshold. **c**, Fine-tuned DreaMS demonstrates superior performance compared to other spectral similarity methods in retrieving molecules from spectral libraries, considering a pool of candidates within a 10 ppm mass difference from the query precursor m/z. Importantly, although zero-shot cosine similarity on DreaMS excels in the correlation metrics presented in **a**, it lacks sensitivity to small structural differences among molecules. This observation motivates us to perform contrastive fine-tuning on challenging examples of molecules with similar masses. **d**, Left: precursor molecules from the fluorine detection test set shown in Fig. 4j. Right: four most similar training examples to the precursor molecules shown on the left. Details on datasets and metrics are provided in Online Methods.

**Extended Data Fig. 6:**
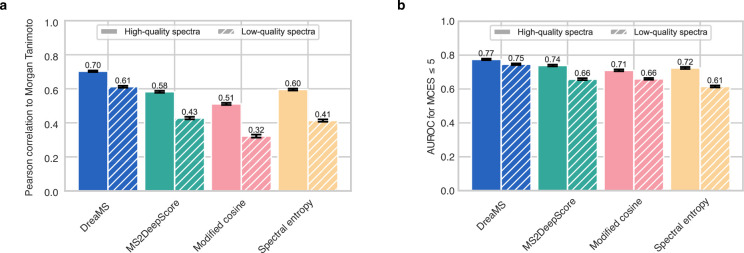
DreaMS embeddings are more robust to the quality of MS/MS spectra than other methods in the context of spectral similarity prediction. Both figures compare the performance of spectral similarity algorithms in terms of pairs where both spectra are either ‘high-quality’ or ‘low-quality’ entries from NIST20. ‘High-quality’ spectra are defined as those satisfying A filtering condition (Fig. 2b; n = 81,699 spectra), while the rest are categorized as ‘low-quality’ (n = 72,861 spectra). Specifically, 98% of low-quality spectra are classified as such because they do not contain at least three peaks with a relative intensity higher than 10%. **a**, The correlation between spectral similarity scores from different algorithms and the Morgan Tanimoto similarities of the corresponding molecular structures (same evaluation setup as in Fig. 4a). **b**, Comparison of the algorithms in the analog search scenario (same as in Fig. 4c) showing the AUROC metric for retrieving molecules with MCES distances less than or equal to five. Both evaluations **a** and **b** demonstrate that the performance drop on low-quality spectra for DreaMS is substantially smaller than for the other methods. Data are presented as mean values with error bars representing 99% confidence intervals, calculated from 1,000 bootstrap samples.

**Extended Data Fig. 7:**
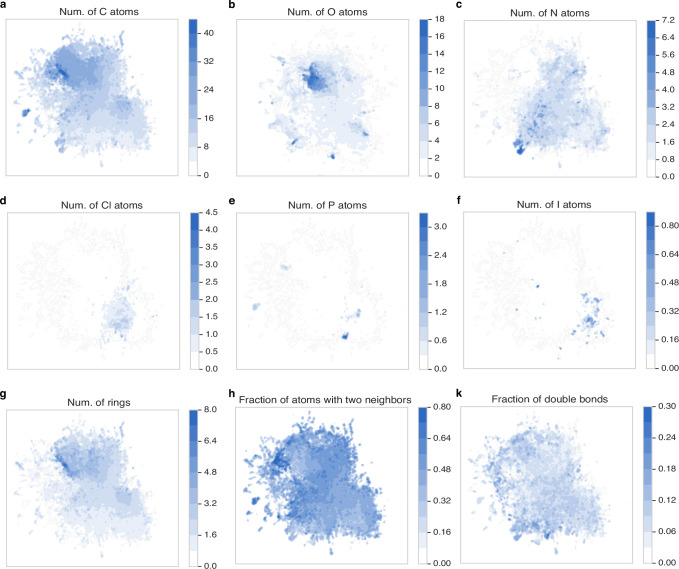
Organization of DreaMS embeddings with respect to various structural properties of molecules. **a**–**f**, UMAP projections of DreaMS embeddings for 100,000 spectra from NIST20 visualized as contour plots colored by the number of atoms of different chemical elements. **g**–**k**, Identical projections as in **a**–**f**, but colored with respect to the topology of molecules. The number of rings and the fraction of atoms with two neighbors characterize the linearity and non-linearity of a molecule. Unlike other depicted properties, the fraction of double bonds is a property depending purely on the connectivity between atoms. Importantly, we show fractions to keep the coloring insensitive to the sizes of molecules, which is typically highly correlated with precursor m/z values of the mass spectra.

The first task that we tackle is spectral similarity, which can be performed directly in the space of DreaMS representations. Remarkably, we observe that, even before any fine-tuning, cosine similarity in the embedding space outperforms the cutting-edge supervised algorithm MS2DeepScore^13^ in terms of correlation with the Tanimoto^65^ and MCES^66^ molecular similarity measures (Fig. 4a). This result emphasizes the amount of information captured by self-supervised representations, especially when considering the fact that MS2DeepScore was explicitly trained on pairs of annotated spectra to approximate their corresponding molecular similarities. Nevertheless, we found that simple zero-shot similarity of DreaMS often lacks sensitivity to small differences in molecular structures (Extended Data Fig. 5b), which are typically crucial for spectral library retrieval and molecular networking. To address this limitation, we disentangle the embeddings of similar molecules through a short but carefully designed contrastive fine-tuning on hard examples. These examples consist of triplets comprising a reference spectrum, a different positive spectrum of the same molecular structure and a negative spectrum of a molecule with a different structure but a similar mass, differing by no more than 0.05 Da from a reference molecule. During fine-tuning, the model refines DreaMS representations by bringing the reference-positive pairs closer together than the reference-negative pairs. We use only a subset of 5,500 molecules from MoNA to avoid biasing the DreaMS representations toward spectral libraries. In a challenging scenario of retrieving similar or different molecules within the 10-ppm precursor *m*/*z* difference, fine-tuned DreaMS substantially outperforms 44 standard spectral similarity measures^9^ (Fig. 4b). The contrastive fine-tuning procedure not only increases sensitivity to details but also globally enhances the correlation with molecular similarities (Fig. 4a and Extended Data Fig. 5a) and the analog search performance (Fig. 4b and Extended Data Fig. 5b), despite not being explicitly optimized for it. Additionally, we found that the resultant embeddings are more robust to the quality of input mass spectra when compared to other methods in the context of spectral similarity (Extended Data Fig. 6). These observations motivated us to use the fine-tuned DreaMS embeddings, rather than the purely self-supervised representations, in all subsequent experiments. The analysis of the resultant embeddings with uniform manifold approximation and projection (UMAP) projections^67^ reveals that they are organized by chemical formulas and structural motifs of the underlying molecules (Fig. 4d and Extended Data Fig. 7). Notably, we found that averaging DreaMS embeddings across samples yields embeddings capturing the composition of complete metabolic profiles (Fig. 5). To our knowledge, there are no existing tools that enable the direct comparison of metabolomes corresponding to different samples or species.

The second problem that we address is predicting Morgan fingerprints from mass spectra and using them to retrieve molecules from PubChem. Notably, in contrast to prior work, our method is capable of predicting fingerprints directly from raw spectra in a single forward pass. This breaks the dependency of machine learning on computationally heavy intermediate steps such as the assignment of chemical formulas to individual input peaks or the combinatorial generation of fragmentation trees. We found that the fine-tuned DreaMS neural network performs on par with the deep learning model MIST in the retrieval of molecular structures using predicted fingerprints (Fig. 4e and Extended Data Table 1), despite the fact that the latter is based on molecular formulas assigned to individual spectral peaks.

The third problem that we tackle is predicting molecular properties of practical interest. Specifically, fast and precise prediction of pharmaceutically relevant chemical properties, such as those involved in Lipinski’s Rule of Five^68^, is essential for the large-scale screening of drug candidates^25^. Similarly, the prediction of Bertz molecular complexity from mass spectra is a promising way to search for biosignatures beyond Earth^26^. The rich molecular knowledge encoded in DreaMS and its fast inference time inspire us to explore the direct prediction of these properties, bypassing the determination of complete molecular structures. We fine-tune the DreaMS neural network to simultaneously predict these and a series of other molecular characteristics. Our model achieves state-of-the-art performance on the prediction of all properties considered for fine-tuning (Fig. 4f,g).

Finally, we address the task of detecting fluorinated molecules from their mass spectra. Currently, there is no practically applicable method capable of detecting fluorine with high precision^69^. This task is particularly challenging because fluorine has only one stable isotope and because fluorinated ions do not exhibit well-defined fragmentation patterns. The state-of-the-art method SIRIUS relies on combinatorial search of fragmentation rules, resulting in a high number of false-positive predictions and requiring extensive runtime. To overcome this limitation, we fine-tune DreaMS to predict the probability of fluorine presence. We evaluate our method on 17,000 previously unreported MS/MS spectra from our in-house library. Whereas SIRIUS does not exceed a precision value of 0.51, DreaMS achieves a precision of 0.91 with a recall of 0.57 and surpasses SIRIUS in recall at low precision values (Fig. 4h). This high precision without a substantial drop in recall on a large test dataset ensures the practical applicability of our method, suggesting that fluorine detections by DreaMS are predominantly correct, and the model confidently identifies half of the fluorinated molecules (Fig. 4i). We additionally demonstrate the strong generalization capacity of our fine-tuned model by identifying correct and confident detection of fluorine for spectra of molecules structurally distinct from all training examples (Fig. 4j).

### DreaMS Atlas—repository-scale molecular network

**Fig. 6:**
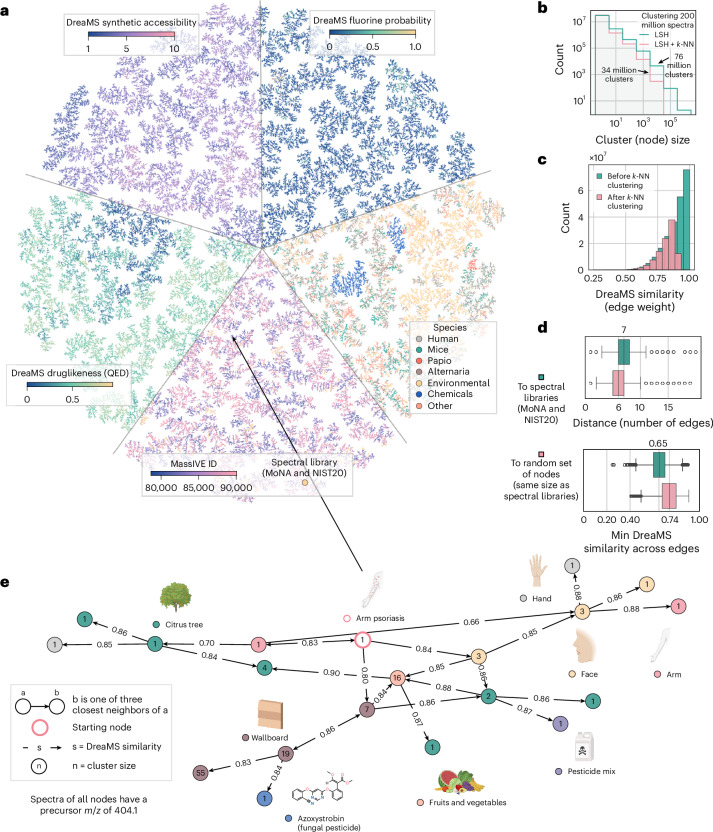
The DreaMS Atlas, a molecular network of 201 million MS/MS spectra, offers a comprehensive systematization of the entire MassIVE GNPS repository. The DreaMS Atlas is built as a three-nearest-neighbor (3-NN) NN-Descent graph^77^ based on DreaMS embedding similarities between MS/MS spectra from GeMS-C1, MoNA and NIST20. Each node includes DreaMS-based property predictions (for example, druglikeness) and MassIVE metadata (for example, species). **a**, TMAP projection^78^ of the 3-NN graph, divided into five pieces, showcasing different node annotations. A subset of 1 million GeMS-C1 nodes and all MoNA and NIST20 nodes are shown. **b**, Each node represents a cluster of mass spectra. First, GeMS-C1 spectra are GeMS-C LSH cluster representatives (green). Second, neighborhoods with DreaMS similarity > 0.9 were collapsed to single nodes (pink). **c**, The DreaMS Atlas is predominantly populated with high-similarity edges, suggesting effective interpolation between spectra of different molecules via transitive connections. Neighborhood clustering eliminated most nearly identical spectra (pink). **d**, Spectral libraries cover the DreaMS Atlas nearly uniformly. The median length of the shortest path from a randomly selected node (*n* = 100,000 random nodes) to the nearest spectral library node is six edges. If spectral libraries are replaced with a random set of nodes of the same size, the median shortest path raises to seven edges (top). However, the difference in median minimum DreaMS similarities along these paths is significant (0.65 versus 0.74; Mann–Whitney *U*-test *P* < 0.05), suggesting that many DreaMS Atlas nodes represent structurally novel molecules relative to spectral library compounds (bottom). Each box plot displays the median (center line), quartiles (box bounds), whiskers extending to 1.5 times the interquartile range and outliers. **e**, Directed three-hop neighborhood of a selected node highlights the DreaMS Atlas as a research hypothesis generator connecting distinct scientific studies. Specifically, a spectrum from the arm psoriasis study links to the spectrum of the fungicide azoxystrobin, suggesting a potential link between psoriasis and the fungicide, abundantly found in various environmental and biological samples. More examples are provided in Extended Data Fig. 8. The icons were created with BioRender.com.

Large-scale metabolomics research is currently constrained by the processing time of spectrum annotation methods. Consequently, the only methods that are practically applicable on a large scale are variations of MASST^51,70^, a traditional modified cosine similarity search algorithm optimized for quickly identifying nearly identical spectra. By contrast, our fully neural network-based models for interpreting MS/MS spectra are both computationally efficient and versatile. Embeddings for 1 million spectra can be computed in approximately 1 h on a machine with an NVIDIA A100 GPU and in about 7 h on a MacBook laptop with an M1 chip. Therefore, we utilize our fine-tuned models to annotate 201 million mass spectra from the MassIVE GNPS repository (covering virtually all positive-mode metabolomics spectra) with DreaMS predictions and organize them into a comprehensive molecular network, which we named the DreaMS Atlas (Fig. 6a).

The DreaMS Atlas is constructed as an approximate three-nearest-neighbor (3-NN) graph based on GeMS mass spectra. Each node represents a DreaMS embedding of a mass spectrum, and each edge represents a DreaMS similarity between the corresponding nodes. To enhance the representativeness and reduce redundancy, we compute the graph for a subset of 34 million mass spectra, which represents 201 million spectra in GeMS-C, clustered based on LSHs and DreaMS similarities (details are provided in Methods and Extended Data Fig. 1b,c). We populate each node with DreaMS molecular property and fluorine presence predictions as well as MassIVE metadata, such as the study descriptions and species information. When constructing the graph, we additionally include nodes corresponding to the embeddings of mass spectra from the MoNA and NIST20 spectral libraries.

Moving on to the analysis of the global composition of the DreaMS Atlas, approximately 33% of nodes represent clusters with more than one spectrum. These clusters are optimized to represent a single molecule (as shown by the precision metric in Extended Data Fig. 1b) or, in the worst-case scenario, multiple molecules with closely related structures (Extended Data Fig. 1c). However, the same structure may potentially be distributed across multiple clusters (recall metric in Extended Data Fig. 1b). Notably, the largest cluster comprises 393,000 spectra from 23,000 distinct LC–MS/MS experiments. The distribution of cluster sizes follows an inverse polynomial trend (Fig. 6b). Regarding edges, the network exhibits strong connectivity, with the majority (67%) of edges displaying high similarities (>0.8), as depicted in Fig. 6c. Simultaneously, 99.7% of the nodes form a single connected component (that is, a subgraph where any pair of nodes can be transitively connected via edges) in the graph, despite the remaining nodes being distributed across 16,000 other connected components. These findings suggest that the DreaMS Atlas enables effective interpolation between spectra of different molecules through strongly connected transitive paths between nodes, even when considering only the three closest neighbors.

This observation motivated us to investigate the connectivity between arbitrary GeMS spectra and spectral library entries. Despite the limited size of the libraries, we found that they are distributed relatively evenly across the DreaMS Atlas. The median length of the shortest path from a randomly selected node to the nearest annotated node from a spectral library is six edges (Fig. 6d, top). If the spectral libraries are replaced with a random set of nodes of the same size as the spectral libraries, the median length of the shortest path raises to seven edges. This observation aligns with the previous findings, which show that spectral libraries cover the space of natural products nearly uniformly^66^. However, the minimum DreaMS similarity along the distance paths is significantly lower for spectral library nodes (0.65 versus 0.74), suggesting that many spectra from MassIVE GNPS represent compounds distinct from the ones present in spectral libraries^52^ (Fig. 6d, bottom).

**Extended Data Fig. 8:**
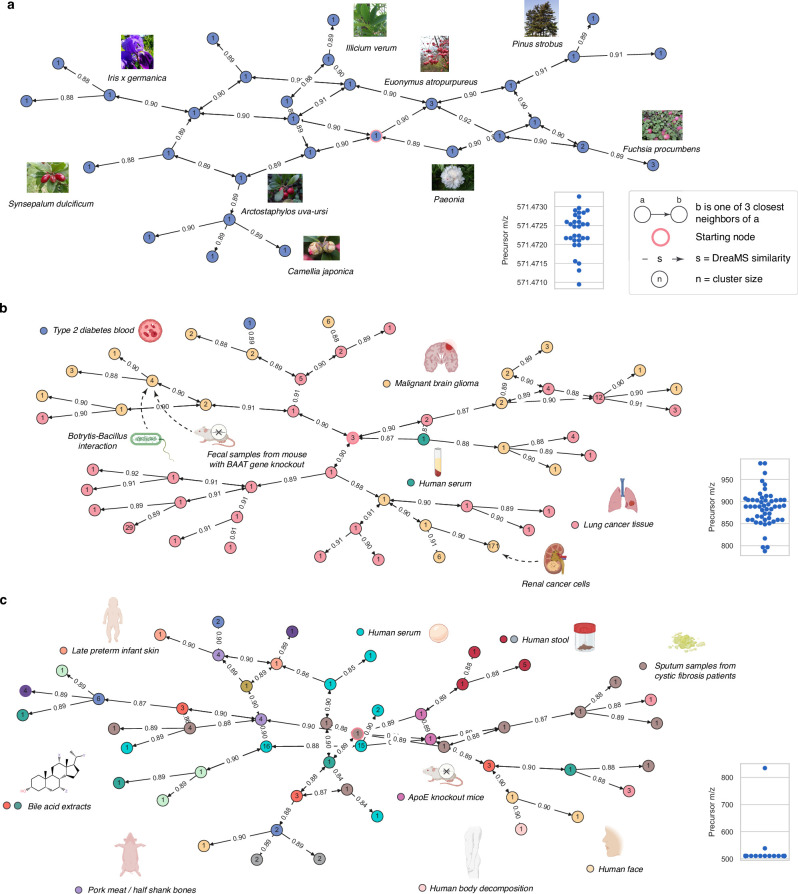
Additional examples of local neighborhoods in the DreaMS Atlas. **a-c**, Each graph illustrates a directed four-hop neighborhood of a randomly selected node (pink node outline) in the DreaMS Atlas. Each node corresponds to a DreaMS embedding of an MS/MS spectrum and represents a cluster of spectra obtained using DreaMS and LSH. Node colors represent distinct studies (that is, MassIVE IDs). Edges point to the nearest neighbors and are labeled with DreaMS similarities. The swarm plots on the right display the precursor m/z values for all nodes. **a**, All spectra originate from the same study characterizing plant tissues but are derived from 34 different plants. They share an identical precursor m/z value up to two decimal places, suggesting that the neighborhood represents a single compound that may be a widely abundant, plant-specific metabolite. **b**, The spectra display a wide variety of precursor m/z values clustered within a single neighborhood. 98% of the spectra originate from three distinct cancer studies conducted in different laboratories: an early-stage lung cancer lipidomics study (pink), a lipidomic analysis of malignant brain gliomas (yellow), and renal cancer cells study (part of a cluster with 171 spectra). This suggests the presence of a family of lipids that may serve as potential cancer biomarkers. Additionally, four other spectra were identified in studies involving type 2 diabetes, microbial interaction, mice with BAAT gene knockout, and human serum, potentially indicating a broader role for these lipids in processes such as inflammation or immune responses. **c**, The spectra in the neighborhood primarily originate from diverse human studies, including research on late preterm infants (light orange), human stool (red, light grey), and human body decomposition (pink). Other spectra come from animal-based studies, such as those on pork meat and ApoE knockout mice, or from bile acid extracts (orange, grey). This suggests that this neighborhood represents a primary bile acid prevalent in the human body across different stages of life, as well as in other organisms. The icons were created using BioRender.

The DreaMS Atlas can serve as a database that allows querying unannotated MS/MS spectra and interpreting them by propagating annotations through their neighbors^71^. We demonstrate this approach using a mass spectrum from an arm psoriasis LC–MS/MS study. Autoimmune diseases such as psoriasis are characterized by complex etiology, which remains incompletely understood^72^. We illustrate how the diversity of the DreaMS Atlas facilitates the exploration of these factors by connecting various scientific studies. Specifically, our analysis reveals a potential association between psoriasis and the fungicide azoxystrobin (Fig. 6e), which, to our knowledge, has not been previously reported. The DreaMS Atlas neighbors also suggest that exposure to azoxystrobin may occur through various environmental sources, such as contaminated food, treated trees or mold- and mildew-resistant wallboards, thereby supporting recent hypotheses regarding the origin of the fungicide in samples from children and pregnant women^73^. Extended Data Fig. 8 provides three additional examples of DreaMS Atlas neighborhoods, presumably representing a plant-specific metabolite abundant across various plant species, a family of lipids potentially serving as cancer biomarkers and a bile acid found in various human samples and other organisms.

## Discussion

Here we introduce DreaMS, a transformer model for interpreting tandem mass spectra. First, we show that, through self-supervised pre-training on GeMS, our large collection of unannotated MS/MS spectra from the GNPS part of MassIVE, the DreaMS neural network, acquires embeddings of mass spectra that reflect underlying molecular structures. Second, we demonstrate the effective fine-tuning capability of DreaMS for a diverse range of mass spectrum annotation problems, achieving state-of-the-art performance across all evaluated tasks. Finally, we present DreaMS Atlas—a comprehensive molecular network constructed using DreaMS annotations for 201 million mass spectra from GeMS.

Although our results strongly indicate the emergence of molecular structure knowledge from training on raw, unannotated mass spectra, the full potential of this approach remains to be unlocked. In particular, we trained our model using only a subset of available mass spectra. Scaling the self-supervised learning to larger datasets, such as by mining spectra from additional repositories such as MetaboLights^74^, and incorporating more diverse mass spectrometry data (for example, including spectra beyond positive ionization mode or singly charged precursor ions) is expected to yield even richer representations of mass spectra, potentially even more accurately capturing the structures of underlying molecules. Additionally, our method is focused solely on tandem mass spectra, disregarding other important features such as MS^1^ isotopic patterns or adduct distributions, which are important, for example, for correct chemical formula determination^23^.

Our work opens up possibilities in two directions of metabolomics-related research. First, we introduce a general data-driven transformer model that can be tailored to virtually any mass spectrum interpretation task, thereby moving away from traditional hand-crafted or rule-based approaches for individual problems. Now that we have made our pre-trained model available to the community, we anticipate that it will provide a starting point (that is, a base model or a feature extractor) for developing more powerful neural network architectures. Second, we introduce the DreaMS Atlas, a comprehensive resource enabling the interpretation of mass spectra by leveraging DreaMS predictions and MassIVE GNPS metadata for 201 million mass spectra. Treating the DreaMS Atlas as an approximation of the space of chemically plausible molecular structures offers new perspectives on various challenges of computational chemistry. For example, fragment-based drug design could be addressed by interpolating between known drugs in the DreaMS Atlas, and the detection of structurally unique compounds with potentially original modes of action can be facilitated by identifying sparsely connected regions in the graph structure of the DreaMS Atlas. Ultimately, annotation of the DreaMS Atlas using a DreaMS model successfully fine-tuned for de novo structure generation has the potential to expand knowledge and understanding of the still largely unexplored chemical space.

## Methods

### Construction of GeMS dataset

To enable self-supervised learning, we mined new large datasets of metabolite MS/MS spectra from the GNPS part of the MassIVE repository, which we named GeMS. MassIVE is a community-driven resource with billions of mass spectra from various biochemical and environmental studies. However, it primarily focuses on proteomics and often contains low-quality data as a result of its uncurated nature. Therefore, we developed a series of algorithms to identify, filter and cluster the metabolomics spectra of MassIVE into high-quality, non-redundant datasets. In this section, we describe our procedure; a more detailed analysis and statistics are available in our technical report^79^.

#### Selecting LC–MS/MS experiments from MassIVE

We start the mining of MassIVE by selecting all .mzML and .mzXML data files from all 4,467 MassIVE datasets (as of November 2022) that are explicitly marked as metabolomics studies with the ‘GNPS’ prefix in their names. This selection yields 338,649 distinct files, among which 249,422 contain MS/MS data with a total of 814 million MS/MS spectra. By filtering out empty or corrupted spectra with invalid *m*/*z* or intensity values (for example, negative intensity or multiple identical *m*/*z* values), we obtain a complete, unprocessed version of GeMS, comprising 714 million MS/MS spectra.

#### Estimating quality of MS data

To obtain higher-quality subsets, we apply file-level and spectrum-level quality criteria to the collected spectra. File-level criteria assess the ordering of spectra based on retention times and tandem MS levels. We discard files with unordered retention times, invalid sequences of MS levels (for example, MS^3^ following MS^1^ without MS^2^), missing MS^1^ data or fewer than three spectra. Notably, we estimate MS instrument accuracy by evaluating the deviation of similar *m*/*z* values within extracted ion chromatograms (XICs). More precisely, the algorithm constructs a set of XICs for MS^1^ base peak masses and then estimates the accuracy of the instrument as the median of standard deviations within individual XICs (Algorithm 1).

The spectrum-level quality criteria operate in several steps. Initially, spectra with a low number of peaks or low intensity amplitudes (that is, the maximum intensity divided by the minimum intensity) are filtered out. Subsequently, non-single charge precursor ions and spectra with excessively high *m*/*z* values (>1,000 Da) are excluded. These steps are crucial for retaining only small metabolite molecules. We keep only spectra acquired in positive ionization mode and filter out those estimated to be non-centroided (Algorithm 2).

##### Algorithm 1 Estimate the absolute accuracy of a mass spectrometry instrument

**Require**: Sequence of MS^1^ spectra from LC–MS experiment.

**Ensure**: Estimated absolute accuracy of mass spectrometry instrument.

 1: *M*_1_ ← M/z values of all base peaks  ⊳ M/z values for 1st round of XICs

 2: *M*_2_ ← {}       ⊳ M/z values for 2nd round of XICs

 3: **for** *m* ∈ *M*_1_ **do**

 4:  *X* ← XIC(*m*, 0.5) ⊳ Set of peaks forming XIC for *m*/*z* *m* and 0.5-Da absolute tolerance

 5:  **if** ∣*X*∣≥5 **then**

 6:   *M*_2_ ← *M*_2_ ∪ MedianMz(*X*)

 7:  **end if**

 8: **end for**

 9: *A* ← {}   ⊳ Accuracy estimates within individual XICs

 10: **for** *m* ∈ *M*_2_ **do**

 11:  *X* ← XIC(*m*, 0.01)       ⊳ XIC with lower 0.01-Da tolerance

 12:  **if** ∣*X*∣≥5 **then**

 13:   *A* ← *A* ∪ StdDevMz(*X*)

 14:  **end if**

 15: **end for**

 16: **return** Median(*A*)

By varying filtering thresholds, we create three GeMS variants: GeMS A (42 million spectra), GeMS B (100 million spectra) and GeMS C (201 million spectra). GeMS A has a low threshold for estimated instrument accuracy (approximately four decimal places in *m*/*z* ratios). GeMS B is primarily filtered by unknown charge values and is less stringent than GeMS A. GeMS C further relaxes criteria applied to GeMS B and is mainly filtered based on criteria related to spectral peak values. Figure 2b provides the details of the applied filters for each subset.

##### Algorithm 2 Estimate the type of a spectrum

**Require**: Spectrum *m*/*z* values ${\bf{m}}\in {{\mathbb{R}}}^{n}$ and intensities ${\bf{i}}\in {{\mathbb{R}}}^{n}$.

**Ensure**: Estimated spectrum type.

 1: **if** *n* < 5 **then**

 2:  **return** CENTROID

 3: **end if**

 4: *b* ← argmax **i**      ⊳ Index of base peak

 5: $$S\leftarrow \{s\in \{1,\ldots, n\}| (\forall {s}^{{\prime} }\in \{0,\ldots, s-b\})({i}_{b+{s}^{{\prime} }} > \frac{{i}_{b}}{2})\}$$

 6: **if** $\max S-\min S < 3$ or ${{\bf{m}}}_{\max S}-{{\bf{m}}}_{\min S} > \frac{\max {\bf{m}}-\min {\bf{m}}}{1000}$ **then**

 7:  **return** CENTROID

 8: **else**

 9:  **if** (∃ *i* ∈ **i**)(*i* = 0) **then**

 10:   **return** PROFILE

 11:  **else**

 12:   **return** THRESHOLDED

 13:  **end if**

 14: **end if**

#### Clustering mass spectra with LSH

The filtering pipeline ensures the quality of individual spectra, but it does not address biases in the entire GeMS datasets related to the natural abundance of metabolites. To tackle this, we employ the random projections algorithm^80^ for efficient clustering and deduplication of mass spectra. This algorithm, falling under the family of LSHs, enables linear-time clustering of MS/MS spectra.

In the first step, we vectorize mass spectra via binning. Specifically, each spectrum is represented as a vector ${\bf{s}}\in {{\mathbb{R}}}^{n}$ with *n* equal-width bins covering the range of *m*/*z* values of interest. The value of **s**_*i*_ then corresponds to the summed intensity of the values contained within the *i-*th bin.

In the subsequent step, for a binned spectrum ${\bf{s}}\in {{\mathbb{R}}}^{n}$, we calculate the corresponding hash *h*(**s**) using a mapping $h:{{\mathbb{R}}}^{n}\to {\{0,1\}}^{m}$ defined as

$$h({\bf{s}})=[{\bf{Ws}}\ge 0],\,\mathrm{where}\,\,{\bf{W}}\in {{\mathbb{R}}}^{m,n}\,\,\mathrm{and}\,\,{{\bf{W}}}_{ij} \sim {\mathcal{N}}(0,1),$$

where [ ⋅ ] indicates an element-wise Iverson bracket, meaning that [*x*_*i*_] = 1 if *x*_*i*_ is true and 0 otherwise. Essentially, each element of the **Ws** product is a dot product of **s** and a random *n*-dimensional hyperplane. Each of the *m* hyperplanes splits the *n*-dimensional space into two complementary subspaces, thereby determining the subspace to which **s** belongs, based on the sign of each dot product. These signs represent the bits of the resulting *m*-dimensional hash. Given that every hyperplane intersects the origin, the likelihood of two binned spectra **s**_*i*_ and **s**_*j*_ sharing the same hash is a function of their cosine similarity^80^:

1 $${\mathbb{P}}\left(h\left({\textbf{s}}_i\right) = h\left({\textbf{s}}_j\right)\right) = 1 - \arccos\left(\underbrace{\frac{{\textbf{s}}_i^{\top}{\textbf{s}}_j}{\|{\textbf{s}}_i\| \|{\textbf{s}}_j\|}}_{{\rm{Cosine}}\,{\rm{similarity}}}\right)\frac{1}{\uppi},$$

where ${\mathbb{P}}$ denotes the joint probability over random hyperplanes. In essence, with a sufficient number of hyperplanes, random projections effectively approximate cosine similarity, which is the primary method for comparing mass spectra.

To cluster the spectra of GeMS, we use *m* = 64 random hyperplanes and the window of size 1 binning the range of *m*/*z* values from 0 to 1,000 Da (that is, *n* = 1,000). By varying the number of retained spectra per cluster, we establish two additional subsets for each of the A, B and C variants of GeMS with, at most, 10 and 1,000 allowed cluster representatives, denoted with additional suffixes such as GeMS-A1 or GeMS-B1000. Figure 2c demonstrates the sizes of the resulting clustered datasets.

#### GeMS data format

We store GeMS datasets in a compressed tensor format using our new .hdf5-based format, primarily designed for deep learning. Supplementary Table 2 outlines the format specifications, detailing all data and metadata entities retained from the input .mzML or .mzXML files.

### Murcko histograms algorithm for splitting molecular datasets

A universal and reliable protocol for supervised learning on spectral libraries is crucial for fine-tuning our pre-trained DreaMS model. The commonly used technique is to split a spectral library into training and validation folds, ensuring that no molecules share identical structures (technically, the first 14 characters of InChI keys) between the folds. However, we identify three issues with this protocol that may limit the generalization capabilities and, therefore, the practical utility of the final fine-tuned model.

First, spectral libraries often contain closely similar structures (ref. ^79^, Section 4.1), such as those resulting from click chemistry. Consequently, molecules with minor structural differences are often assigned to different training/validation folds, introducing a data leakage for tasks such as fingerprint prediction, where small structural details may not substantially impact performance metrics. Second, structure-disjoint splits are agnostic to the fragmentation nature of MS/MS. For instance, two molecules differing only in the length of the carbon chain connecting two subfragments have distinct structures, yet such chains can be easily fragmented by CID, resulting in nearly identical spectra. Third, the structure-disjoint approach often assigns entire molecules and their abundant fragments (such as the fragments of sugars) to different folds, increasing the chance of overfitting to abundant substructures. To address these issues, we designed a new algorithm, Murcko histograms, based on the Murcko scaffolds^64^, for splitting molecular structures into training/validation folds.

#### Algorithm 3 Definition of a Murcko histogram

**Require**: Molecular graph *G* = (*V*, *E*), *V* = {1, …, *n*}, *E* ⊆ {{*u*, *v*}∣*u*, *v* ∈ *V* ∧ *u* ≠ *v*}.

**Ensure**: Murcko histogram *h*.

 1: *G* ← MurckoScaffold(*G*)

 2: *V*_*R*_ ← {*V*_*r*_ ⊂ *V* ∧ ∣*V*_*r*_∣ > 3∣*V*_*r*_ contains all atoms of a (fused) ring}

 3: *V*_*L*_ ← {*v* ∈ *V*∣deg(*v*) > 1 ∧ *v* is not in any ring}

 4: *h* ← a map ${{\mathbb{N}}}^{2}\to {\mathbb{N}}$ initialized as $(\forall i,j\in {{\mathbb{N}}}^{2})(h(i,j)=0)$

 5: **for** *V*_*r*_ ∈ *V*_*R*_ **do**

 6:  $$r\leftarrow \sum \{| {V}_{r}\cap {V}_{r}^{{\prime} }/2| | {V}_{r}^{{\prime} }\in {V}_{R}\setminus {V}_{r}\}$$

 7:  *l* ← ∣*V*_*r*_ ∩ *V*_*l*_∣

 8:  *h*(*r*, *l*) ← *h*(*r*, *l*) + 1

 9: **end for**

 10: **return** *h*

To address the first issue, we built our method upon coarse-grained Murcko scaffolds. To tackle the second issue of insensitivity to fragmentation, our method operates on molecular fragments as the primary design principle. To address the third issue, we define a heavily relaxed notion of molecular similarity, ensuring that the distinction between folds is well defined.

In particular, our algorithm computes a histogram defined in terms of the counts of scaffold substructures (Algorithm 3). Given the Murcko scaffold of a molecule^64^, the algorithm operates on two separate groups of its atoms. The first group consists of sets of atoms, with each set determining a ring (line 2 in the algorithm), whereas the second group includes all atoms connecting these rings (that is, linkers; line 3). For each ring, the algorithm calculates a pair of natural numbers: the number of neighboring rings and the number of adjacent linkers (denoted as *r*, *l* in lines 5–9). These pairs define the domain of the resulting histogram, where the values represent the counts of such pairs within a molecule (lines 4, 10). Extended Data Fig. 4a shows examples of Murcko histograms and the corresponding molecular structures.

The Murcko histogram-disjoint training/validation splitting resolves the first two aforementioned issues by being insensitive to minor atomic details and by taking into account the fragments of molecular scaffolds instead. We further address the third issue by defining a way to compare the histograms that is more relaxed than a simple identity (Algorithm 4). Specifically, we define a distance on Murcko histograms as the difference in the histogram values solely in rings, not considering the number of neighboring linkers. Using this definition, we relocate the samples from validation to training folds if their distance is less than 5 and not performing the relocation if the minimum number of rings in one of the molecules is less than 4. Notice that these parameters provide interpretability for the boundary between training and validation folds, and, by varying them, we can balance between the number of validation examples and the degree of similarity between training and validation folds in terms of scaffold substructures.

Unlike structure-disjoint splitting, our method eliminates virtually all near-duplicate training/validation leaks, resulting in a two-fold reduction in average Morgan Tanimoto similarity^65^ between the molecules corresponding to training and validation spectra (Extended Data Fig. 4b).

With this approach, we define approximately 90%/10% training/validation splits for MoNA (25,319/3,557 spectra corresponding to 5,524/831 molecules) as well as for the combined MoNA and NIST20 datasets (439,927/43,040 spectra corresponding to 25,476/2,454 molecules). These splits are used to fine-tune the pre-trained DreaMS model, and, throughout the text, we refer to them as Murcko histogram-disjoint splits. As mentioned previously, the name originates from the use of Murcko scaffolds^64^ as the basis for the algorithm. We anticipate that our training/evaluation protocol based on Murcko histograms will stimulate further research into the development of a new generation of models with enhanced generalization toward the undiscovered dark metabolome^5^.

#### Algorithm 4 Definition of a Murcko subhistogram relation

**Require**: Two Murcko histograms *h*_1_ and *h*_2_, a minimum number of rings *k* to compute the non-identity relation and a minimum difference in ring-only Murcko histogram *m* to consider the histograms different. The default values are *k* = 4 and *m* = 5.

**Ensure**: True if one of *h*_1_, *h*_2_ is a subhistogram of the other in Murcko rings, False otherwise.

 1: **if** $$\min \{{\sum }_{i,j\in {\mathbb{N}}}{h}_{1}(i,j),{\sum }_{i,\;j\in {\mathbb{N}}}{h}_{2}(i,j)\} < k$$ **then**

 2:  **return** *h*_1_ = *h*_2_

 3: **end if**

 4: $$d\leftarrow {\sum }_{i\in {\mathbb{N}}}| ({\sum }_{j\in {\mathbb{N}}}{h}_{1}(i,j)-{\sum }_{j\in {\mathbb{N}}}{h}_{2}(i,j))|$$

 5: **if** *d* < *m* **then**

 6:  **return** True

 7: **else**

 8:  **return** False

 9: **end if**

### DreaMS neural network architecture

The DreaMS neural network architecture (Fig. 3b) can be decomposed into three main consecutive modules. Given a mass spectrum, the model first encodes each spectral peak into a high-dimensional continuous representation with PeakEncoder. Then, it processes the entire set of encoded peaks with SpectrumEncoder—a series of transformer encoder blocks^58^. Each block learns relationships between peaks and consecutively enriches their representations. The final task-specific PeakDecoder adjusts the final transformer representations according to a task-specific training objective. Each of the modules is described in detail below.

#### PeakEncoder

We represent each raw mass spectrum as a matrix ${\bf{S}}\in {{\mathbb{R}}}^{2,n+1}$, constructed as

2 $${\bf{S}}=\left[\begin{array}{ccccc}{m}_{0}&{m}_{1}&{m}_{2}&\ldots \,&{m}_{n}\\ 1.1&{i}_{1}&{i}_{2}&\ldots \,&{i}_{n}\end{array}\right],$$

where each column, indexed by *j* ∈ {1, …, *n*}, corresponds to one of the *n* (we set *n* = 60 for pre-training and *n* = 100 for fine-tuning) spectral peaks and is represented as the continuous vector ${[{m}_{j},{i}_{j}]}^{\top }\in {\mathbb{R}}\times [0,1]$, denoting the pair of *m*/*z* and relative intensity values (*m*/*z* denoted by *m* and intensity denoted by *i*). Additionally, we prepend a precursor *m*/*z* *m*_0_ and assign it an artificial intensity of 1.1. We term this additional peak the precursor token and utilize it as a master node^57^ for aggregating spectrum-level information. If a spectrum has more than *n* peaks, we select the *n* most intense ones; if it has fewer than *n* peaks, we pad the matrix **S** with zeros.

Rather than treating each *m*/*z* ratio as a single continuous value, we process it using a mass-tolerant modification of Fourier features $\Phi :{\mathbb{R}}\to {[-1,1]}^{2B}$ (ref. ^59^), dependent on *B* predefined frequencies ${\bf{b}}\in {{\mathbb{R}}}^{B}$. Specifically, the features are constructed with sine and cosine functions

3 $$\Phi {(m)}_{i}=\sin (2\uppi {b}_{i}m),\qquad \Phi {(m)}_{i+1}=\cos (2\uppi {b}_{i}m),$$

where each frequency *b*_*i*_ is uniquely associated with either a low frequency capturing the integer part of a mass $m\in {\mathbb{R}}$ or a high frequency capturing its decimal part, forming together a vector of frequencies

4 $${\mathbf{b}} = \left[\underbrace{\frac{1}{m_{\rm{max}}}, \frac{1}{m_{\rm{max}} - 1}, \dots}_{{\rm{Low}}\,{\rm{frequencies}}}, \frac{1}{1}, \underbrace{\frac{1}{k m_{\rm{min}}}, \frac{1}{(k - 1) m_{\rm{min}}}, \dots, \frac{1}{ m_{\rm{min}}}}_{{\rm{High}}\,{\rm{frequencies}}} \right]^{\top} \in {\mathbb{R}}^B.$$

Here constants ${m}_{\min }\in (0,1)$ and ${m}_{\max }\in (1,\infty )$ represent the minimum decimal mass of interest (that is, the absolute instrument accuracy) and the maximum integer mass of interest, and $k\in {\mathbb{N}}$ is such that *km*_min_ is the closest value to 1. For instance, when training DreaMS on GeMS-A spectra, we set *m*_min_ = 10^−4^ and *m*_max_ = 1,000 according to the construction of GeMS datasets. This schema yields 1,000 low frequencies and 5,000 high frequencies (that is, the overall dimensionality of the vector **b** is 6,000).

Furthermore, we process the Fourier features given by equation (3) with a feed-forward neural network ${{\rm{FFN}}}_{F}:{{\mathbb{R}}}^{2B}\to {{\mathbb{R}}}^{{d}_{m}}$ (*d*_*m*_ = 980 in our final model). We hypothesize that the sensitivity of Fourier features to both large and small differences in masses allows FFN_*F*_ to learn the space of plausible molecular masses given by elemental compositions. Our instantiation of frequencies outperforms both random initialization^59^ and the log-spaced sinusoidal variant proposed for proteomics^14,81^ (Extended Data Fig. 3; ref. ^79^). Notably, because peaks do not form a sequence of tokens but, rather, a set, we do not encode their positions, in contrast with classic positional encoding^58^.

The concatenation of the output of FFN_*F*_ with the output of another shallow feed-forward network ${{\rm{FFN}}}_{P}:{{\mathbb{R}}}^{2}\to {{\mathbb{R}}}^{{d}_{p}}$ (*d*_*p*_ = 44 in our final model) applied to raw *m*/*z* and intensity values forms the complete PeakEncoder: ${{\mathbb{R}}}^{2}\to {{\mathbb{R}}}^{{d}_{m}+{d}_{p}}$:

5 $${\rm{PeakEncoder}}(m,i)={{\rm{FFN}}}_{F}(\Phi (m))\,\parallel \,{{\rm{FFN}}}_{P}(m,i),$$

where ∥ denotes concatenation. Column-wise application of PeakEncoder to the matrix **S** yields a high-dimensional representation of the corresponding spectrum ${{\bf{S}}}_{0}\in {{\mathbb{R}}}^{d,n}$, where *d* = *d*_*m*_ + *d*_*p*_ (*d* = 1,024 in our final model) is the dimensionality of the representation, and *n* is the number of peaks.

#### SpectrumEncoder

Given the output of PeakEncoder, SpectrumEncoder: ${{\mathbb{R}}}^{d,n}\to {{\mathbb{R}}}^{d,n}$ updates the representations of peaks by exchanging information between individual peaks via the self-attention mechanism. This is achieved through a sequence of *l* (*l* = 7 in our final model) transformer encoder layers (that is, BERT^43^), alternating multi-head self-attention blocks with peak-wise feed-forward networks. Starting from **S**_0_, each *i*-th block gradually updates the representation of the spectrum from **S**_*i*−1_ to **S**_*i*_. Throughout the text, we denote the columns of **S**_*l*_ (that is, representations of individual peaks) as **s**_0_, …, **s**_*n*_. We refer to the first columns of such matrices, representing precursor tokens, as DreaMS.

An important property of the transformer encoder is its equivariance to permutations of tokens^82^. Combined with the position-invariant encoding of peaks through PeakEncoder, this implies that the same two peaks in different spectra will have identical attention scores in the first attention layer, regardless of the total number of peaks or noise signals between these two peaks. Multiple related works in metabolomics and proteomics have employed the transformer architecture to encode mass spectra^25,28,32,83–85^. To further strengthen the inductive bias of the transformer toward the relations between peaks, we explicitly enrich the attention mechanism with all pairwise *m*/*z* differences including neutral losses. In each transformer layer, the attention score **A**_*ij*_ between the *i*-th and *j*-th peaks is computed as:

6 $${{\bf{A}}}_{ij}=\frac{{{\bf{q}}}_{i}^{\top }{{\bf{k}}}_{j}+\mathop{\sum }\nolimits_{k}^{2t}\Phi {({m}_{i})}_{k}-\Phi {({m}_{j})}_{k}}{\sqrt{d}},$$

where ${{\bf{q}}}_{i}^{\top }{{\bf{k}}}_{j}$ is a standard dot-product attention, and $\mathop{\sum }\nolimits_{k}^{2t}\Phi {({m}_{i})}_{k}-\Phi {({m}_{j})}_{k}$ is an additional Graphormer-like term^61^. Element-wise differences in Fourier features enable the transformer to directly attend to precise *m*/*z* differences, enhancing its capacity to learn fragmentation patterns and robustness to shifts in absolute *m*/*z* values. This is particularly important, for instance, in scenarios where *m*/*z* values are shifted due to the masses of ionization adducts. We use eight attention heads per transformer block in our final model.

In contrast to BERT, we use a pre-norm variant of transformer^86^, remove biases in linear layers and use ReLU activations. We utilize the implementation of transformer provided by Nguyen et al.^87^.

#### PeakDecoder

Depending on the training objective, we use linear layers of different shapes (referred to as heads) to refine and project the final hidden representations of peaks given by the SpectrumEncoder.

For both *m*/*z* masking and retention order pre-training objectives, we employ simple linear projections followed by suitable activation functions, mapping the representations of peaks into the corresponding domains of predictions:

7 $${\hat{{\bf{y}}}}_{{\rm{mass}}}={\rm{softmax}}\left({{\bf{W}}}_{{\rm{mass}}}{{\bf{s}}}_{k}\right),\qquad {\hat{y}}_{{\rm{order}}}=\sigma \left({{\bf{W}}}_{{\rm{order}}}\left({{\bf{s}}}_{0}^{(i)}\parallel {{\bf{s}}}_{0}^{(\!j)}\right)\right),$$

where ${{\bf{s}}}_{k}\in {{\mathbb{R}}}^{d}$ denotes the hidden representation of a masked peak *k* ∈ *M* from a set of masked indices *M* ⊂ {1, …, *n*}. It is projected by ${{\bf{W}}}_{{\rm{mass}}}\in {{\mathbb{R}}}^{c,d}$ and the Softmax function to obtain the predicted probability vector ${\hat{{\bf{y}}}}_{{\rm{mass}}}\,\in \,{{\mathbb{R}}}^{c}$ with *c* classes corresponding to the discretized mass bins to be reconstructed. Next, ${\hat{y}}_{{\rm{order}}}$ denotes the predicted probability that a spectrum *i* precedes the spectrum *j* in chromatography. The probability is predicted by concatenating two precursor embeddings ${{\bf{s}}}_{0}^{(i)},{{\bf{s}}}_{0}^{(\!j)}$ corresponding to the two spectra and applying the linear projection ${{\bf{W}}}_{{\rm{order}}}\in {{\mathbb{R}}}^{1,2d}$ followed by the sigmoid function *σ*.

For supervised fine-tuning tasks, we employ two variants of linear heads. The first variant is given by single linear layers operating solely on the precursor token representations ${{\bf{s}}}_{0}\in {{\mathbb{R}}}^{d}$:

8 $${\hat{{\bf{y}}}}_{{\rm{props}}}={{\bf{W}}}_{{\rm{props}}}{{\bf{s}}}_{0},\qquad {\hat{y}}_{{\rm{F}}}=\sigma ({{\bf{W}}}_{{\rm{F}}}{{\bf{s}}}_{0}),\qquad {\bf{z}}={{\bf{W}}}_{{\rm{emb}}}{{\bf{s}}}_{0},$$

where ${{\bf{W}}}_{{\rm{props}}}\in {{\mathbb{R}}}^{11,d}$, ${{\bf{W}}}_{{\rm{F}}}\in {{\mathbb{R}}}^{1,d}$ followed by sigmoid *σ* and ${{\bf{W}}}_{{\rm{emb}}}\in {{\mathbb{R}}}^{d,d}$ yield the predictions of 11 molecular properties ${\hat{{\bf{y}}}}_{{\rm{props}}}$, the probability of fluorine presence ${\hat{y}}_{{\rm{F}}}$ and the spectral embedding **z**, respectively.

For the task of predicting molecular fingerprints, we find a head with richer representation capacity to slightly improve the performance:

9 $${\hat{{\bf{y}}}}_{{\rm{fp}}}={{\bf{W}}}_{{\rm{fp1}}}\mathop{\sum }\limits_{i=0}^{n}{\rm{ReLU}}({{\bf{W}}}_{{\rm{fp0}}}{{\bf{s}}}_{i}).$$

Here the projections ${{\bf{W}}}_{{\rm{fp0}}}\in {{\mathbb{R}}}^{d,d}$ and ${{\bf{W}}}_{{\rm{fp1}}}\in {{\mathbb{R}}}^{4096,d}$ are arranged into the DeepSets-like^88^ head to output 4,096 fingerprint elements. In this case, the head operates on the hidden representations of all peaks **s**_*i*_ rather than solely on the precursor peak **s**_0_ as in equation (8). The details of pre-training and fine-tuning objectives are discussed in the following sections.

### Self-supervised pre-training

The objective of self-supervised pre-training for DreaMS is defined by minimizing a weighted sum of two losses:

10 $${{\mathcal{L}}}_{{\rm{DreaMS}}}=0.8{{\mathcal{L}}}_{{\rm{mass}}}+0.2{{\mathcal{L}}}_{{\rm{order}}},$$

where ${{\mathcal{L}}}_{{\rm{mass}}}$ represents the masked modeling loss, quantifying the error of the model in reconstructing the masses of randomly masked peaks, and ${{\mathcal{L}}}_{{\rm{order}}}$ denotes the retention order prediction error. Each training example within a mini-batch consists of sampling two spectra with indices *i*, *j* from the same LC–MS/MS experiment. Here, we further detail the computation of both ${{\mathcal{L}}}_{{\rm{mass}}}$ and ${{\mathcal{L}}}_{{\rm{order}}}$ losses for the example pair *i*, *j*.

To compute the ${{\mathcal{L}}}_{{\rm{mass}}}$ loss, we randomly sample a predefined ratio (30%) of peaks *M*^(*i*)^, *M*^(*j*)^ ⊂ {1, …, *n*} from both spectra *i* and *j*, proportionally to their intensities. Then, we replace the masses of the sampled peaks in the spectra with −1.0, while keeping the intensities unchanged, and utilize the original mass values ${{\bf{m}}}^{(i)}\in {{\mathbb{R}}}^{| {M}^{(i)}| }$ and ${{\bf{m}}}^{(\!j)}\in {{\mathbb{R}}}^{| {M}^{(\!j)}| }$ as the prediction labels. Instead of directly predicting the continuous values **m**^(*i*)^, **m**^(*j*)^, we categorize them into *c* equal-width bins ranging from 0 to the maximum *m*/*z* of the training dataset (1,000 Da for GeMS-A subsets; Fig. 2b) and train the model to predict the correct bins. This classification approach^89^, rather than regression, is adopted to better capture the inherent uncertainty of mass reconstruction, as it accounts for the possibility that several masses may be equally plausible for a masked peak. A regression model may converge at predicting the average value, whereas a classification model would learn to assign equal probability to each plausible mass.

Specifically, we convert continuous mass values into degenerate categorical distributions, represented by binary matrices ${{\bf{Y}}}_{\rm{mass}}^{(i)}\in {\{0,1\}}^{| {M}^{(i)}|, c}$ and ${{\bf{Y}}}_{\rm{mass}}^{(\!j)}\in {\{0,1\}}^{| {M}^{(\!j)}|, c}$, where rows correspond to masked peaks and columns correspond to mass bins. The elements of the matrices are ones in bins containing the corresponding masses and zeros elsewhere. In detail, for a masked peak *l* ∈ *M*^(*k*)^ in spectrum *k* ∈ {*i*, *j*} and bin *b* ∈ {0, …, *c* − 1}, the corresponding matrix element is

11 $${y}_{{\rm{mass}},l,b}^{(k)}=\left[{m}_{l}^{(k)}\in \left[b\frac{1,000}{c},(b+1)\frac{1,000}{c}\right)\right],$$

where [ ⋅ ] indicates the Iverson bracket, implying [*x*] = 1 if *x* is true and 0 otherwise. The terms $\frac{1,000}{c}$ represent the *m*/*z* range (0, 1,000) discretized into *c* bins. We set *c* = 20,000 in our final model.

Then, the model is trained to predict a categorical distribution for each of the masked peaks ${\hat{{\bf{Y}}}}_{{\rm{mass}}}^{(i)},{\hat{{\bf{Y}}}}_{{\rm{mass}}}^{(\!j)}$ (equation (7), left), and the reconstruction error is evaluated using the cross-entropy loss in the space of discretized mass values:

12 $${{\mathcal{L}}}_{{\rm{mass}}}\left({\hat{{\bf{Y}}}}_{{\rm{mass}}}^{(i)},{{\bf{Y}}}_{{\rm{mass}}}^{(i)},{\hat{{\bf{Y}}}}_{{\rm{mass}}}^{(\!j)},{{\bf{Y}}}_{{\rm{mass}}}^{(\!j)}\right)=-\frac{1}{2}\sum _{k\in \{i,\,j\}}\sum _{l\in {M}^{(k)}}{{{\bf{y}}}_{{\rm{mass}},l}^{(k)}}^{\top }\log \left({\hat{{\bf{y}}}}_{{\rm{mass}},l}^{(k)}\right),$$

where the first sum from the left averages the results across two sampled spectra *i* and *j*, and the second sum iterates over all masked peaks *M*^(*k*)^ in spectrum *k*. The dot-product ${{{\bf{y}}}_{{\rm{mass}},l}^{(k)}}^{\top }\log ({\hat{{\bf{y}}}}_{{\rm{mass}},l}^{(k)})$ calculates the cross-entropy between a ground-truth degenerate distribution ${{\bf{y}}}_{{\rm{mass}},l}^{(k)}$, which contains a 1 for the correct mass bin of peak *l* in spectrum *k* and 0s elsewhere, and the corresponding predicted distribution over bins ${\hat{{\bf{y}}}}_{{\rm{mass}},l}^{(k)}$. Minimizing ${{\mathcal{L}}}_{\rm{mass}}$ effectively maximizes the likelihood of predicting the correct mass bins, and the loss is minimal when all the bins are predicted correctly.

The second component of the DreaMS loss, ${{\mathcal{L}}}_{{\rm{order}}}$, is given by a binary cross-entropy classification loss. The model is trained to predict the retention order of two spectra *i* and *j* within the LC–MS/MS experiment by estimating the probability ${\hat{y}}_{{\rm{order}}}$ that spectrum *i* precedes spectrum *j* in chromatography (equation (7), right). The actual probability *y*_order_ is either 0 or 1:

13 $${{\mathcal{L}}}_{{\rm{order}}}=-\left(\;{y}_{{\rm{order}}}\log\left({\hat{y}}_{{\rm{order}}}\right)+\left(1-{y}_{{\rm{order}}}\right)\log \left(1-{\hat{y}}_{{\rm{order}}}\right)\right).$$

We pre-train DreaMS on the GeMS-A10 dataset and retain the 60 highest peaks when forming training batches. Additionally, with a 20% probability, we augment a spectrum by adding a random scalar from (0, 50) to all its *m*/*z* values. Such modification forces the neural network to learn relationships between spectral peaks rather than memorizing precise masses, a property important for making the model more robust to, for example, different ionization adducts.

#### Linear probing of the emergence of molecular structures

Every 2,500 pre-training iterations, we conduct linear probing—a technique enabling us to evaluate the gradual emergence of molecular structures during self-supervision. Specifically, we freeze a model and train a single linear layer ${{\bf{W}}}_{{\rm{probe}}}\in {{\mathbb{R}}}^{166,d}$, followed by a sigmoid activation function (that is, a logistic regression) to predict 166 MACCS fingerprint bits from precursor token embeddings, utilizing a random subsample of 6,000 examples from the Murcko histogram split of NIST20 and MoNA. We employ a binary cross-entropy loss function (equation (13)) for learning individual fingerprint bits. We select MACCS fingerprints as the probing objective because they offer an interpretable description of a molecular structure, allowing each predicted bit to be reconstructed back to a molecular substructure.

We report the average validation recall in predicted bits as a function of pre-training time (Fig. 3c) to illustrate the model’s progressively improving ability to discover the substructures of ground-truth molecules. For each iteration, we display the highest recall within 100 probing epochs. Notably, although the figure depicts only the increase in recall, this improvement is achieved without any decline in precision. In fact, precision slightly increases from 0.81 to 0.84 within the same evaluation setup.

#### Related work in supervised learning on mass spectra

The self-supervised pre-training objectives introduced in our work, namely masked *m*/*z* prediction (equation (13)) and retention order prediction (equation (13)), are related to the supervised tasks of spectrum simulation, MS/MS intensity prediction and retention time prediction given a molecular structure. However, because DreaMS does not utilize molecular structures as input, there are considerable differences between our approach and the corresponding supervised methods. First, spectrum simulation or intensity prediction methods are typically trained to reconstruct complete MS/MS spectra via dot-product-based regression loss functions^20,21,90,91^. In contrast, our approach focuses on reconstructing only a fraction of randomly sampled *m*/*z* values using a cross-entropy loss. Second, retention order prediction is generally treated as a molecular property prediction, often formulated as a regression task to predict retention times^91–93^. Because we do not assume knowledge of molecular structures, we train DreaMS to classify which spectrum elutes first in chromatography by sampling two spectra from the same LC–MS/MS experiment.

The primary goal of self-supervised pre-training is to obtain embeddings of MS/MS spectra that can be utilized for various downstream applications. Prior works derived embeddings in a supervised manner by employing contrastive learning approaches. For instance, GLEAMS derives embeddings of peptide MS/MS spectra and applies them for downstream clustering^15^. Goldman et al.^32^ investigated the MS/MS spectra embeddings derived by the supervised contrastive MIST model in the context of chemical classes of small molecules.

### Transfer learning to spectrum annotation tasks

In this section, we discuss how we transfer the knowledge obtained by the DreaMS model during the self-supervised pre-training to make predictions in scenarios of practical interest. Specifically, we describe how we fine-tune the pre-trained model end to end (that is, fine-tuning all parameters in the PeakEncoder and SpectrumEncoder layers) for various downstream mass spectrometry tasks, using task-specific heads instead of PeakDecoder.

#### Spectral similarity

The cosine similarity on unsupervised DreaMS embeddings exhibits a strong correlation with Tanimoto similarity (Fig. 4a). However, we observe that it lacks sensitivity to small structural differences among molecules with nearly identical masses (Extended Data Fig. 5b). To address this limitation, we refine the embedding space through contrastive fine-tuning. Specifically, we utilize triplet margin loss function^94^ to disentangle the embeddings of spectra that share similar molecular masses:

14 $${{\mathcal{L}}}_{{\rm{emb}}}({\bf{z}},{{\bf{z}}}^{+},{{\bf{z}}}^{-})=\max \{\cos ({\bf{z}},{{\bf{z}}}^{+})-\cos ({\bf{z}},{{\bf{z}}}^{-})+{\it{\Delta}}, 0\},$$

where ${\bf{z}}\in {{\mathbb{R}}}^{d}$ denotes the embedding of a randomly sampled reference spectrum, **z**^+^; ${{\bf{z}}}^{-}\in {{\mathbb{R}}}^{d}$ are the embeddings of positive and negative examples, respectively; and *Δ* > 0 (*Δ* = 0.1 in our final setting) is the contrastive margin. The positive example is defined as a spectrum of the same molecule as the reference spectrum (having the same 14-character prefix in the InChI key), whereas the negative example is given by a spectrum corresponding to a different molecule but with a similar molecular mass (at most 0.05-Da difference). The ${{\mathcal{L}}}_{\rm{emb}}$ loss function optimizes the embedding space so that the reference spectra are closer to the positive examples than to the negative ones. The contrastive margin *Δ*, intuitively, measures the minimum required gap between the corresponding positive and negative distances. The proximity between two embeddings **a** and **b** is measured by cosine similarity:

15 $$\cos ({\bf{a}},{\bf{b}})=\frac{{{\bf{a}}}^{\top }{\bf{b}}}{\max \{\parallel {\bf{a}}\parallel \parallel {\bf{b}}\parallel, \epsilon \}},$$

where *ϵ*, set to 10^−8^, is a constant for numerical stability.

The aim of the fine-tuning is to adjust the embedding space using minimal supervision yet still retaining the knowledge acquired during self-supervised pre-training and not introducing biases of spectral libraries scarcity. Therefore, we conduct contrastive training on a refined subset of MoNA histogram-disjoint split containing approximately 25,000 spectra corresponding to 5,500 unique InChI connectivity blocks and do not use any spectra from NIST20 for training. To form a subset, we retain only the spectra satisfying A quality conditions (as shown in Fig. 2b), having [M+H]^+^ adducts and 60-eV collision energy. To simulate the performance evaluation on a new spectral library, we evaluate the cosine similarity in refined embedding space on the high-quality subset of NIST20 satisfying A filtering conditions. We additionally exclude from the validation all NIST20 examples whose InChI key connectivity blocks are present in MoNA. We consider three molecular similarity tasks: estimating the Tanimoto similarity between Morgan fingerprints of underlying molecules, determining the spectra corresponding to the same molecules within the pool of candidate spectra with similar precursor masses and search for structural analogs of the precursor molecules of a query spectrum.

Specifically, in the case of the Tanimoto similarity approximation problem, we measure Pearson correlation between DreaMS cosine similarities and Tanimoto similarities on binary Morgan fingerprints (number of bits = 4,096, radius = 2) using approximately 82,000 pairs of spectra sampled from NIST20 so that they maximize the entropy of the distribution of ground-truth similarities. We benchmark our method against the official implementation (https://github.com/matchms/ms2deepscore) of the state-of-the-art MS2DeepScore model^13^ (as depicted in Fig. 4a).

For the second task of retrieving mass spectra corresponding to the same molecule, we measure the area under the receiver operating characteristic curve (AUROC), which evaluates the classification performance under different similarity thresholds. We sample approximately 750,000 binary classification examples from NIST20 in a way that makes positive class examples correspond to pairs of spectra having the same underlying molecular structure (as measured by the same 14-character prefix in the InChI key) and negative class examples correspond to pairs of spectra having similar precursor masses (with, at most, 10-ppm precursor *m*/*z* difference). We benchmark our method against spectral entropy, the state-of-the-art method, as well as 43 other baseline approaches^9^ (as illustrated in Fig. 4b). We use the implementation of all the methods from the official spectral entropy GitHub repository (https://github.com/YuanyueLi/SpectralEntropy).

For the third task of analog search, we utilize the same dataset as used for the Tanimoto similarity benchmark but measure a different metric. Specifically, we base this metric on the maximum common edge subgraph (MCES)^66^, which expresses the edit distance on molecular graphs. This metric approximates the number of structural changes, thereby allowing us to formalize the notion of structural analogs as molecules with an MCES distance of less than or equal to a certain MCES threshold *k*. We compare the analog search performance of DreaMS, DreaMS (zero-shot), MS2DeepScore, modified cosine similarity and spectral entropy using the AUROC, with true labels defined as pairs of spectra whose molecules have an MCES distance of less than or equal to *k* ∈ {0, 1, …, 7} (Fig. 4c and Extended Data Fig. 5b).

For the visualization of fine-tuned embeddings (Figs. 4d and 5 and Extended Data Fig. 7), we utilize the UMAP algorithm^67^, with cosine similarity set as the metric. Figure 4d and Extended Data Fig. 7 display 100,000 random embeddings of NIST20 spectra, with all precursor InChI keys disjoint from the precursors of the MoNA subset used for the spectral similarity fine-tuning. Level set plots in Extended Data Fig. 7 present 10 levels of various molecular properties when binning the UMAP axes into 200 bins. Sample-average embeddings in Fig. 5 are computed for 2,810 food samples (that is, .mzML files; 6 million spectra in total) from the MSV00008490 dataset (https://massive.ucsd.edu/ProteoSAFe/dataset.jsp?task=ce3254fe529d43f48077d7ad55b7da09), which have textual food descriptions assigned in the metadata table within the dataset repository. We assigned color categories to individual data points using the ChatGPT-4 model^76^. Specifically, we provided the model with a list of all unique sample descriptions and queried the model with the following prompt: ‘Please summarize all the individual textual descriptions into the minimal number of categories, including a ‘Miscellaneous’ category’. We then iteratively asked the model to further reduce the number of categories by merging related ones (for example, combining ‘Cheese’ and ‘Dairy products’ into ‘Cheese & Dairy Products’). After obtaining 13 categories, we excluded all 948 samples forming the ‘Miscellaneous’ category, such as those containing ‘supplement’ or ‘extract’ in their descriptions.

#### Molecular fingerprint prediction

The next problem that we tackle with DreaMS is the prediction of molecular fingerprints. We adapt our model via supervised fine-tuning and validate it on the MIST CANOPUS benchmark^32^ to evaluate the performance against the state-of-the-art deep learning model MIST.

In detail, we fine-tune DreaMS to directly predict molecular fingerprints via the cosine similarity loss function ${{\mathcal{L}}}_{{\rm{fp}}}$ between true **y**_fp_ and predicted ${\hat{{\bf{y}}}}_{{\rm{fp}}}$ fingerprints:

16 $${{\mathcal{L}}}_{{\rm{fp}}}\left({\hat{{\bf{y}}}}_{{\rm{fp}}},{{\bf{y}}}_{{\rm{fp}}}\right)=\cos \left({\hat{{\bf{y}}}}_{{\rm{fp}}},{{\bf{y}}}_{{\rm{fp}}}\right),$$

where cos is the cosine similarity given by equation (15), discussed previously in the context of comparing embeddings of spectra.

For the fine-tuning and evaluation, we use the CANOPUS benchmark from the official GitHub repository (https://github.com/samgoldman97/mist). Specifically, we use the MIST codebase to generate fingerprints and the candidate pools of molecules for the evaluation. Each pool corresponds to a single spectrum along with positive and negative candidate molecules mined from PubChem. The positive candidates correspond to molecules in PubChem that have the same 14-character prefix in the InChI key as the true underlying molecule, including the true molecule itself. The negative candidates are given by the molecules sharing the same molecular formula. Then, the retrieval performance is evaluated using the accuracy at top *k* metrics for *k* ∈ {1, 5, 10, 20, 50, 100, 200}, measuring the number of spectra that have at least one positive molecule in the top *k* predictions, sorted by the cosine similarity between the predicted and ground-truth fingerprints (Fig. 4e and Extended Data Table 1).

It is worth noting that the MIST CANOPUS benchmark is limited in size, covering 8,000 spectra from 7,000 unique molecules. Given this fact and the recent assessment of MIST’s generalization capabilities after being trained on the MIST CANOPUS dataset^95^, the fine-tuning of DreaMS to predict molecular fingerprints should be understood as a proof of concept that our approach can achieve competitive performance with MIST without relying on domain-specific components (for example, SIRIUS peak formula annotations), rather than as a production-ready fingerprint predictor.

#### Molecular property prediction

Next, we fine-tune DreaMS to predict molecular properties. For this, we reproduce the evaluation protocols proposed previously by Voronov et al.^25^ and Gebhard et al.^26^.

Specifically, we fine-tune our model to jointly predict *r* = 11 selected molecular properties from spectra, averaging the squared error for each of the properties:

17 $${{\mathcal{L}}}_{{\rm{props}}}({\hat{{\bf{y}}}}_{{\rm{props}}},{{\bf{y}}}_{{\rm{props}}})=\frac{1}{r}\parallel {\hat{{\bf{y}}}}_{{\rm{props}}}-{{\bf{y}}}_{{\rm{props}}}{\parallel }^{2},$$

where ${{\bf{y}}}_{{\rm{props}}}\in {{\mathbb{R}}}^{r}$ denotes the vector containing ground-truth molecular properties, such as quantitative estimation of drug-likeness (QED), synthetic accessibility and Bertz complexity (see Fig. 4 for the complete list). Because different properties have different scales and are measured in different units, we normalize them before feeding them to the loss function. In particular, we map each property to the [0, 1] interval via min-max scaling based on the statistics from the training data.

For the training, validation and testing, we use the MoNA and NIST20 dataset splits prepared using our Murcko histograms algorithm. First, inspired by Gebhard et al.^26^, we evaluate the performance of DreaMS on predicting molecular complexity from mass spectra. In detail, we estimate the capability of DreaMS to predict the Bertz complexity of a molecule from its mass spectum, by measuring its relative prediction error under different minimum true complexity thresholds of interest. The relative prediction error is defined as $| \,{y}_{{\rm{Bertz}}}-{\hat{y}}_{{\rm{Bertz}}}| /{y}_{{\rm{Bertz}}}$ and measures the performance of predicting complexity ${\hat{y}}_{{\rm{Bertz}}}$ robustly under varying absolute values of the true complexity *y*_Bertz_^26^. We compare our method against XGBoost^26,96^ trained on 10,000-dimensional binned spectra with 0.1-Da bin size and the state-of-the-art spectra property predictor MS2Prop^25^, reimplemented and retrained to predict Bertz complexity among other properties (Fig. 4f). We also evaluate our method and XGBoost on predicting ten other properties addressed by MS2Prop (Fig. 4g). Our reimplementation of MS2Prop uses the hyperparameters described in the original publication^25^ and the same values as DreaMS for the unspecified hyperparameters (such as batch size and learning rate).

#### Fluorine detection

We evaluate the performance of DreaMS on detecting fluorinated molecules from mass spectra.

Our fluorine detector is fine-tuned using a binary cross-entropy loss function ${{\mathcal{L}}}_{{\rm{F}}}$ with additional focal loss terms^97^ accounting for class imbalance. For each training example, the loss is computed as:

18 $${{\mathcal{L}}}_{{\rm{F}}}\left(\,{\hat{y}}_{{\rm{F}}},{y}_{{\rm{F}}}\right)=-{\alpha }_{\rm{F}}{\left(1-{p}_{{\rm{F}}}\right)}^{\gamma }\log {p}_{{\rm{F}}},$$

where ${\hat{y}}_{{\rm{F}}}$ is the predicted fluorine presence probability, and *y*_F_ is the 0 or 1 label, depending on the ground-truth presence of fluorine. Next, *p*_F_ is the standard binary cross-entropy term, and *α*_F_ and *γ* are focal loss terms:

19 $${p}_{\rm{F}}=\left\{\begin{array}{ll}{\hat{y}}_{{\rm{F}}},\quad &{\rm{if}}\,{y}_{{\rm{F}}}=1\\ 1-{\hat{y}}_{{\rm{F}}},\quad &{\rm{otherwise}},\end{array}\right.\qquad {\alpha }_{{\rm{F}}}=\left\{\begin{array}{ll}\alpha, \quad &{\rm{if}}\,{y}_{{\rm{F}}}=1\\ 1-\alpha, \quad &{\rm{otherwise}},\end{array}\right.$$

where *α* = 0.8 increases the loss for underrepresented examples, containing fluorine, and decreases the loss otherwise (training data contain approximately 80% of examples with fluorine); *γ* = 0.5 adjusts the predicted probabilities of correct classes to prioritize misclassified examples.

We fine-tune DreaMS on the spectra from MoNA and NIST using the Murcko histograms algorithm for training/validation splitting. Subsequently, we test the performance of the model on our in-house dataset, consisting of 17,052 [M+H]^+^ Orbitrap mass spectra (3,900 spectra of 1,175 unique fluorinated molecules and 13,152 spectra of 4,055 unique non-fluorinated molecules), by measuring precision and recall under different thresholds (Fig. 4h). The dataset is available under MassIVE accession number MSV000094528 (https://massive.ucsd.edu/ProteoSAFe/dataset.jsp?task=676a38e2dd574a15905e807d78cf1e57). As a baseline, we use SIRIUS 5.6.3 with the possible adducts set to [M+H]^+^, the instrument to Orbitrap and the maximum number of fluorine elements to 5 (maximum number in the dataset)^30^. By experimenting with the numbers lower than 5, we observe a substantial drop in recall but no improvement in precision.

We prioritize high precision over recall as we find it the most practically important metric when searching for new fluorinated molecules for further wet-lab characterization, considering the difficulty of wet-lab experiments. Consequently, we estimate the coverage of mass spectra with confident predictions using the model operating in the high-precision regime with the precision of 90%. Specifically, we set two predicted probability thresholds (0.46 and 0.75) for classifying spectra containing and not containing fluorine so as to lend the model 90% precision in both cases. Notably, we find that only 5% of spectra have uncertain predictions (with the predicted probabilities in the [0.46, 0.75] interval), whereas the rest of the spectra are covered by high-confidence predictions (Fig. 4i).

### DreaMS Atlas

In this section, we provide a description of how the DreaMS Atlas is constructed. We start by outlining the process of selecting and annotating nodes for the DreaMS Atlas, followed by the process of connecting the nodes to form a graph structure.

The construction process begins with generating DreaMS embeddings for 76 million spectra comprising GeMS-C1 subset of the GeMS dataset. This subset represents LSH cluster representatives of 201 million GeMS-C spectra, covering the entire MassIVE GNPS repository. Spectra from blank samples, identified by specific suffixes in their names (for example, ‘blank’, ‘no_inj’, ‘noinj’, ‘empty’, ‘solvent’ or ‘wash’), are excluded. Additionally, we enrich individual nodes with DreaMS molecular property and fluorine presence predictions, along with relevant metadata obtained from the MassIVE repository, such as information about the study species, respective study description and the instrument used for spectrum acquisition. Finally, we include embeddings of mass spectra from the MoNA and NIST20 spectral libraries. To avoid redundancy in the spectral libraries with respect to molecular structures, we merge spectra sharing identical canonical SMILES but differing in adduct species from both MoNA and NIST20, resulting in 79,000 merged spectra from 819,000 library entries.

Next, we employ the NN-Descent algorithm^77^ to compute an approximate 3-NN graph, where nodes represent DreaMS embeddings and edges represent similarities between these embeddings. To further refine the LSH clustering, 3-NN neighborhoods sharing DreaMS similarities above 0.9 are clustered into single nodes, and the *k*-NN graph is reconstructed for 34 million nodes representing the clusters. More precisely, to cluster the nodes, we iterate over all nodes sorted in descending order by their degrees and run a breadth-first search (BFS) from each node. The BFS stops if either an edge has a DreaMS similarity smaller than 0.9 or the DreaMS similarity between the starting node and the new candidate node is smaller than 0.9. All the nodes aggregated through the BFS are collapsed to a single cluster and are represented by a starting node. This algorithm allows us to cluster the graph in linear time. It is worth noting that, by defining neighborhoods based on similarity thresholds rather than the number of hops, this algorithm adjusts the graph topology, preventing over-representation of certain spectra.

This procedure results in the creation of a final 3-NN graph representing the DreaMS Atlas. We utilize the PyNNDescent implementation of NN-Descent by McInnes et al. (https://github.com/lmcinnes/pynndescent), which provides functionalities for managing the vector database of the *k*-NN graph, such as querying the graph with new DreaMS embeddings not present in the DreaMS Atlas or extending the DreaMS Atlas with new embeddings.

### Benchmarking of MS/MS clustering with LSH and DreaMS embeddings

Throughout our work, we employed three techniques to cluster MS/MS spectra: LSH, DreaMS *k*-NN aggregation and *k*-NN classification in the principal component analysis (PCA) space of DreaMS embeddings. This section provides details on each of the techniques and an assessment of their clustering quality.

To reduce redundancy in the GeMS dataset and create a more balanced representation of the underlying molecular structures, we employed an LSH clustering technique. The LSH technique works by comparing the hashes of binned spectra and has two key parameters: the *m*/*z* bin width and the number of LSH hyperplanes. Extended Data Fig. 1a illustrates the performance of LSH clustering with varying *m*/*z* bin widths across different numbers of hyperplanes. The metrics include the mean intra-cluster modified cosine similarity, the mean precursor *m*/*z* standard deviation per cluster, the dataset size (expressed as the fraction of clusters relative to the original dataset size) and LSH computation time. These metrics were computed using 94,837 spectra from the MassSpecGym dataset^98^, specifically the spectra of [M+H]^+^ ions with at least three signals having relative intensities greater than 10%. As the number of hyperplanes increases, LSH clusters show improved consistency in approximating cosine similarity and maintaining precursor *m*/*z* values while retaining more unclustered spectra. Interestingly, the *m*/*z* bin width has a minor effect on the resulting clusters but substantially impacts the algorithm’s running time.

For constructing the GeMS subsets, we used LSH clustering with 30 hyperplanes and an *m*/*z* bin width of 1.0. The leftmost group of bars in Extended Data Fig. 1b presents the performance of this LSH configuration for clustering precursor molecules. The metrics shown include precision and recall based on the 2D InChI key identities, along with the dataset size after clustering.

By design, LSH is a high-precision, low-recall algorithm. To improve recall for constructing the DreaMS Atlas, we combined multiple LSH clusters into new larger clusters. This was achieved using a BFS aggregation in the *k*-NN graph of the DreaMS Atlas, applying a specific DreaMS similarity cutoff to form new clusters. As shown in Extended Data Fig. 1b, a similarity cutoff of 0.9 increases recall while maintaining a median precision of 1 (the second group of bars). Although lower cutoffs yield higher recall, they also increase the rate of false positives. To ensure that each node in the DreaMS Atlas represents a single molecular structure, we used the 0.9 cutoff. Extended Data Fig. 1c provides an example of a cluster refined using DreaMS *k*-NN with a 0.9 cutoff, which results in the precision lower than 1.0. The cluster contains 30 spectra, 28 of which correspond to the same structure, whereas two are different but highly similar. Nevertheless, all the structures are highly similar and exhibit highly similar, potentially indistinguishable, MS/MS spectra.

The third clustering technique, *k*-NN classification in the PCA space of DreaMS embeddings, was used to explore the organization of the self-supervised DreaMS embedding space. Our goal was to evaluate the linearity of the embedding space with respect to the underlying molecular structures. To do so, we clustered the embeddings in affine PCA subspaces of varying dimensionality, grouping *k* nearest neighbors into individual clusters, and evaluated their molecular composition. Extended Data Fig. 1d generalizes Fig. 3e and quantitatively demonstrates the linear organization of self-supervised DreaMS representations with respect to molecular structures, thus highlighting their robustness to mass spectrometry conditions. The figure shows *k*-NN majority voting accuracy for 2D InChI keys in the DreaMS embedding space of NIST20 spectra. Nearest neighbors were defined using cosine similarity, and the accuracy was estimated using 100,000 random spectra. In the affine PCA subspace of 1,023 dimensions (embedding dimensionality minus 1), accuracy reaches 0.865 for *k* = 3 and 0.999 for *k* = 1, showcasing the linearity of the embedding space in grouping spectra from identical compounds. In other words, the embeddings are clustered according to molecular structures, even in the linear subspace of the original embedding space.

### Hyperparameters, ablation studies, implementation details and benchmarking

We report the hyperparameters used for pre-training and fine-tuning in Supplementary Tables 3 and 4, respectively. Extended Data Fig. 3 summarizes the key ablation studies for self-supervised pre-training, highlighting most important features of our method. First, pre-training on the high-quality GeMS-A10 subset of GeMS, containing 24 million MS/MS spectra, results in better performance compared to training on larger but lower-quality subsets, such as GeMS-A1000 or GeMS-B, which include up to 100 million spectra (Extended Data Fig. 3a). Additionally, we found that retaining 60 peaks (as the sequence length for the transformer) instead of 30 leads to improved performance, emphasizing the importance of lower-intensity peaks beyond the most intense ones.

Second, pre-processing *m*/*z* values with mass-tolerant Fourier features and subsequently passing them through a high-capacity feed-forward network substantially enhances performance. For instance, tokenizing *m*/*z* values with binning decreases performance, similar to using only two feed-forward layers instead of four or employing random Fourier features or sinusoidal embeddings instead of our pre-defined mass-tolerant sine and cosine waves (Extended Data Fig. 3b). Other important components of the architecture include relatively large 1,024-dimensional embeddings and *m*/*z* shift augmentations.

Third, framing the masked peak prediction task as classification, rather than regression, has a critical impact on the quality of the resulting embeddings (Extended Data Fig. 3c). Although other design choices, such as the fraction of masked peaks, predicting retention orders or masking peaks based on their intensities, contribute to performance improvements, they are not as impactful as using the cross-entropy classification loss function.

For both pre-training and fine-tuning, we used the Adam optimizer with default parameters^99^. All models were trained using either four AMD MI250X GPUs or eight NVIDIA A100 GPUs in a distributed data parallel (DDP) mode. The final DreaMS model was pre-trained for 48 h, and its fine-tuning runtime never exceeded several hours. With 8 NVIDIA A100 GPUs, the generation speed of embeddings (that is, forward pass through the trained model) averages approximately 1.2 ± 0.002 million embeddings per hour, where the standard deviation is calculated based on 12 chunks comprising 79 million mass spectra from GeMS-C1.

We used matchms^100^ and pyOpenMS^101^ Python libraries for processing mass spectra. All neural networks were implemented in PyTorch^102^ and trained using PyTorch Lightning^103^.

We plan to continuously report the performance of more advanced fine-tuning protocols developed in future work within the MassSpecGym benchmarking environment^98^.

### Reporting summary

Further information on research design is available in the Nature Portfolio Reporting Summary linked to this article.

## Online content

Any methods, additional references, Nature Portfolio reporting summaries, source data, extended data, supplementary information, acknowledgements, peer review information; details of author contributions and competing interests; and statements of data and code availability are available at 10.1038/s41587-025-02663-3.

## Supplementary information

Supplementary Information Supplementary Tables 1–4.

Reporting Summary

## Data Availability

The GeMS dataset, DreaMS Atlas and publicly available labeled MS/MS data used for fine-tuning can be downloaded from our Hugging Face Hub repository^104^. The pre-trained model weights are hosted on Zenodo^105^. Our in-house data for fluorine detection evaluation are available under MassIVE accession number MSV000094528 (ref. ^106^), and the food datasets are available at MSV000094528 (ref. ^107^). The MoNA spectral library can be downloaded from the official website^108^, whereas the NIST20 library is not publicly available due to licensing restrictions. The MassSpecGym dataset, used to evaluate MS/MS clustering performance and DreaMS attention heads, can be downloaded from the official Hugging Face repository^109^.

## Extended data

**Extended Data Table 1:** Full test metrics on MIST PubChem retrieval benchmark

Method	Top 1	Top 5	Top 10	Top 20	Top 50	Top 100	Top 200	Cos. similarity
FFN fingerprint	17.309	37.121	45.611	54.634	63.946	71.329	77.359	0.537
FFN contrastive	20.632	44.996	54.389	63.536	75.062	80.558	86.095	-
MIST fingerprint	29.368	55.332	63.536	72.231	80.476	85.726	89.418	0.695
MIST contrastive	28.384	55.373	65.217	72.970	81.255	85.480	89.377	-
MIST contrastive + fingerprint	30.703	58.120	68.927	75.709	84.094	87.916	92.355	-
DreaMS fingerprint (ours)	32.731	59.352	67.719	75.390	82.404	87.121	90.771	0.646

The ‘Top k’ columns stand for the retrieval accuracy@*k* metrics reported in percents (higher is better). ‘Cos. similarity’ shows the cosine similarity (higher is better) between predicted and ground-truth fingerprints (that is, inverted test loss). Methods with the ‘fingerprint’ suffix denote the methods directly predicting molecular fingerprints, whereas ‘contrastive’ means that the training procedure involves batches of PubChem molecules with the same molecular formula and learns to correctly rank candidates via a noise contrastive estimation (NCE) loss function^32^. We do not experiment with the contrastive extension for our model because it outperforms contrastive methods on top 1 and top 5 metrics without considering additional PubChem molecules. Interestingly, DreaMS underperforms MIST in terms of the cosine similarity despite performing better in retrieval. A similar performance on the same benchmark is observed with SIRIUS, which outperforms MIST in retrieval despite being less accurate in fingerprint prediction^32^.

## Code availability

The DreaMS codebase is publicly available on our GitHub repository^110^ and has been permanently archived on Zenodo^111^. The corresponding documentation and tutorials are available at the Read the Docs page^112^.
