# Supplementary material for: Self-supervised learning of molecular representations from millions of tandem mass spectra using DreaMS
Source: Nat Biotechnol. 2025 May 23;44(4):630–40. doi: 10.1038/s41587-025-02663-3 (PMC13090125; doi:10.1038/s41587-025-02663-3)
Supplement: Supplementary file 1 — Supplementary Tables 1–4. [file 41587_2025_2663_MOESM1_ESM.pdf]

nature biotechnology

Supplementary information https://doi.org/10.1038/s41587-025-02663-3

Self-supervised learning of molecular representations from millions of tandem mass spectra using DreaMS

In the format provided by the authors and unedited

Supplementary information: Self-supervised learning of molecular representations from millions of tandem mass spectra using DreaMS

Roman Bushuiev[1,2†], Anton Bushuiev[2†], Raman Samusevich[1,2], Corinna Brungs[1], Josef Sivic[2*] and Tomáš Pluskal[1*]

[1]Institute of Organic Chemistry and Biochemistry of the Czech Academy of Sciences.
[2]Czech Institute of Informatics, Robotics and Cybernetics, Czech Technical University.

*Corresponding author(s). E-mail(s): josef.sivic@cvut.cz; tomas.pluskal@uochb.cas.cz;
Contributing authors: roman.bushuiev@uochb.cas.cz; anton.bushuiev@cvut.cz;
†These authors contributed equally to this work.

Filter	GeMS-A	GeMS-B	GeMS-C
Total num. spectra	711,449,200	711,449,200	711,449,200
Est. instr. accuracy > 1e-4/1e-3/1e-3	128,986,494	618,134,504	618,134,504
Negative polarity	105,515,251	550,518,161	550,518,161
Unk. charge/Unk. charge/-	80,127,413	198,921,862	550,518,161
Charge > 1	73,734,411	170,443,646	522,039,945
Precursor m/z > 1000/1500/1500	73,517,815	169,869,998	520,791,059
MS level > 2/2/10	73,478,267	169,357,985	520,791,059
Max m/z > 1000/1500/1500	73,444,038	166,526,375	459,817,491
Estimated as profile spectrum	72,579,055	165,334,485	455,106,642
Num. of intense (> 10%) peaks < 3	59,123,728	128,761,221	353,491,125
Intensity amplitude < 20/20/18	43,137,912	101,062,679	202,409,915
Num. of spectra after filtering < 3	43,137,657	101,062,396	202,409,083

Table S1 Summary of the GeMS data-quality filtering pipeline. Each value represents the number of spectra remaining after applying a specific filter (row) to a particular GeMS subset (column). Slashes (i.e., "/") in the filters separate different settings for the GeMS-A, GeMS-B, and GeMS-C subsets, ordered according to the column order.

MS^2 data	Data type
M/z values	float64
Intensities	float32
MS level	int8
RT	float32
Charge	int8
Polarity	int8
Precursor m/z	float32
Window lbound	float32
Window ubound	float32
CID energy	float32
Spectrum type	int8
Ion injection time	float32
Definition string	utf-8 str
Precursor id	int32

Metadata	Data type
File name	utf-8 str
Instrument name	utf-8 str
MS level order	utf-8 str
$\|X_1\|$	int64
$\|X_2\|$	int64
$\textsc{Median}(A)$	float64

Precursor data	Data type
M/z values	float64
Intensities	float32
RT	float32
Ion injection time	float32
Id	int32

Table S2 Specification of the GeMS `.hdf5` data format. "MS^2 data" and "Precursor data" are `.hdf5` groups whereas "Metadata" entities are `.hdf5` attributes. All tensors are one-dimensional of the length equal to the number of collected spectra. The only exception is "M/z values" and "Intensities" which are two-dimensional arrays of the number of spectra by the number of peaks shape. We retain 128 highest peaks and pad the array with zeros. $|X_1|$, $|X_2|$, and $\textsc{Median}(A)$ correspond to the intermediate values and the output of the algorithm estimating the absolute accuracy of a mass spectrometry instrument (Algorithm 1 in Online Methods). "Window lbound" and "Window ubound" correspond to the lower and upper bounds of the MS^1 isolation window. "Definition string" is a spectrum metadata summary string available in the data from Thermo instrument.

**Hyperparameter**	**Values**
Learning rate	$5 \cdot 10^{-5}, 9 \cdot 10^{-5}, \mathbf{1 \cdot 10^{-4}}, 2 \cdot 10^{-4}, 3 \cdot 10^{-4}$
Number of warmup steps [1]	0, **5000**, 20000
Batch size	1024, 2048, **4096**
Number of transformer layers l	1, 5, **7**, 11
Number of attention heads	4, **8**, 12, 16
Transformer hidden dimensionality d	512, 768, **1024**
Fourier features dimensionality d_m	24, 512, **980**
Peak dimensionality d_p	**24**, 512, 980
FFN_F depth	2, 4, **5**
FFN_F hidden dimensionality	256, **512**
FFN_P depth	**1**, 2, 3
Attention mechanism	dot-product, additive [2], **Graphormer**
Dropout	0.0, **0.1**, 0.5
Weight decay	**0.0**, $1 \cdot 10^{-5}$
Fraction of masked peaks	0.1, 0.2, **0.3**, 0.4, 0.5
Mask sampling strategy	uniform (intensity $>$ 10%), **intensity proportional**
Deterministic mask sampling	**True**, False
Retention order loss weight	0.0, **0.2**, 0.5
Focal loss γ	0, 0.5, 2, **5**
Dataset	**GeMS-A10**, GeMS-A1000, GeMS-A, GeMS-B
Training float precision	**32 bits**, 64 bits

Table S3 Explored pre-training hyperparameters. The optimal values, used for the extraction of embeddings, zero-shot predictions, and further fine-tuning, are highlighted in bold.

Spectral similarity

Hyperparameter	Values
Learning rate	$3 \cdot 10^{-6}, \mathbf{5 \cdot 10^{-6}}, 1 \cdot 10^{-5}$
Batch size	**32**, 64
Head type	**Linear**
Triplet margin Δ	0.05, **0.1**, 0.2, 0.5

Molecular fingerprint prediction

Hyperparameter	Values
Learning rate	$2 \cdot 10^{-5}, 3 \cdot 10^{-5}, \mathbf{4 \cdot 10^{-5}}$
Batch size	16, **32**, 64
Head type	Linear, **DeepSets**

Molecular property prediction

Hyperparameter	Values
Learning rate	$3 \cdot 10^{-4}, \mathbf{3 \cdot 10^{-5}}$
Batch size	128, **512**
Head type	**Linear**

Fluorine detection

Hyperparameter	Values
Learning rate	$3 \cdot 10^{-5}, \mathbf{5 \cdot 10^{-5}}$
Batch size	64, **128**
Head type	**Linear**
Focal loss α	0.5, 0.6, **0.8**
Focal loss γ	**0.5**, 1, 2

Table S4 Explored fine-tuning hyperparameters. The optimal values, used for test predictions, are highlighted in bold.

References

[1] Vaswani, A. *et al.* Guyon, I. *et al.* (eds) *Attention is all you need.* (eds Guyon, I. *et al.*) *Advances in Neural Information Processing Systems 30: Annual Conference on Neural Information Processing Systems 2017, December 4-9, 2017, Long Beach, CA, USA*, 5998–6008 (2017). URL https://proceedings.neurips.cc/paper/2017/hash/3f5ee243547dee91fbd053c1c4a845aa-Abstract.html.

[2] Song, J., Kim, S. & Yoon, S. Moens, M., Huang, X., Specia, L. & Yih, S. W. (eds) *Alignart: Non-autoregressive neural machine translation by jointly learning to estimate alignment and translate.* (eds Moens, M., Huang, X., Specia, L. & Yih, S. W.) *Proceedings of the 2021 Conference on Empirical Methods in Natural Language Processing, EMNLP 2021, Virtual Event / Punta Cana, Dominican Republic, 7-11 November, 2021*, 1–14 (Association for Computational Linguistics, 2021). URL https://doi.org/10.18653/v1/2021.emnlp-main.1.
