# Supplementary material for: Self-supervised learning of molecular representations from millions of tandem mass spectra using DreaMS
Source: Nat Biotechnol. 2025 May 23;44(4):630–40. doi: 10.1038/s41587-025-02663-3 (PMC13090125; doi:10.1038/s41587-025-02663-3)
Supplement: Supplementary file 2 — Reporting Summary [file 41587_2025_2663_MOESM2_ESM.pdf]

nature portfolio

Corresponding author(s): Tomáš Pluskal, Josef Sivic

Last updated by author(s): Mar 19, 2025

Reporting Summary

Nature Portfolio wishes to improve the reproducibility of the work that we publish. This form provides structure for consistency and transparency in reporting. For further information on Nature Portfolio policies, see our Editorial Policies and the Editorial Policy Checklist.

Statistics

For all statistical analyses, confirm that the following items are present in the figure legend, table legend, main text, or Methods section.

n/a	Confirmed	
☐	☒	The exact sample size (*n*) for each experimental group/condition, given as a discrete number and unit of measurement
☒	☐	A statement on whether measurements were taken from distinct samples or whether the same sample was measured repeatedly
☒	☐	The statistical test(s) used AND whether they are one- or two-sided<br>*Only common tests should be described solely by name; describe more complex techniques in the Methods section.*
☒	☐	A description of all covariates tested
☒	☐	A description of any assumptions or corrections, such as tests of normality and adjustment for multiple comparisons
☐	☒	A full description of the statistical parameters including central tendency (e.g. means) or other basic estimates (e.g. regression coefficient) AND variation (e.g. standard deviation) or associated estimates of uncertainty (e.g. confidence intervals)
☒	☐	For null hypothesis testing, the test statistic (e.g. F, t, r) with confidence intervals, effect sizes, degrees of freedom and P value noted<br>*Give P values as exact values whenever suitable.*
☒	☐	For Bayesian analysis, information on the choice of priors and Markov chain Monte Carlo settings
☒	☐	For hierarchical and complex designs, identification of the appropriate level for tests and full reporting of outcomes
☒	☐	Estimates of effect sizes (e.g. Cohen's *d*, Pearson's *r*), indicating how they were calculated

Our web collection on statistics for biologists contains articles on many of the points above.

Software and code

Policy information about availability of computer code

Data collection	The DreaMS codebase is publicly available on our GitHub repository (https://github.com/pluskal-lab/DreaMS) and has been permanently archived on Zenodo (https://zenodo.org/records/13843034). A list of specific versions of all the Python packages used can be found in the setup.py file (https://github.com/pluskal-lab/DreaMS/blob/main/setup.py). Documentation and tutorials for the codebase are accessible on our Read the Docs page (https://dreams-docs.readthedocs.io).
Data analysis	The DreaMS codebase is publicly available on our GitHub repository (https://github.com/pluskal-lab/DreaMS) and has been permanently archived on Zenodo (https://zenodo.org/records/13843034). A list of specific versions of all the Python packages used can be found in the setup.py file (https://github.com/pluskal-lab/DreaMS/blob/main/setup.py). Documentation and tutorials for the codebase are accessible on our Read the Docs page (https://dreams-docs.readthedocs.io).

For manuscripts utilizing custom algorithms or software that are central to the research but not yet described in published literature, software must be made available to editors and reviewers. We strongly encourage code deposition in a community repository (e.g. GitHub). See the Nature Portfolio guidelines for submitting code & software for further information.

Data

Policy information about availability of data

All manuscripts must include a data availability statement. This statement should provide the following information, where applicable:

- Accession codes, unique identifiers, or web links for publicly available datasets
- A description of any restrictions on data availability
- For clinical datasets or third party data, please ensure that the statement adheres to our policy

The GeMS dataset, DreaMS Atlas, and publicly available labeled MS/MS data used for fine-tuning can be downloaded from our Hugging Face Hub repository (https://huggingface.co/datasets/roman-bushuiev/GeMS/tree/main). The pre-trained model weights are hosted on Zenodo (https://zenodo.org/records/10997887). Our in-house data for fluorine detection evaluation is available under the MassIVE accession number MSV000094528 (https://massive.ucsd.edu/ProteoSAFe/dataset.jsp?task=676a38e2dd574a15905e807d78cf1e57), and the food datasets are available at MSV00008490 (https://massive.ucsd.edu/ProteoSAFe/dataset.jsp?task=ce3254fe529d43f48077d7ad55b7da09). The MoNA spectral library can be downloaded from the official website (https://mona.fiehnlab.ucdavis.edu/), while the NIST20 library is not publicly available due to licensing restrictions. The MassSpecGym dataset, used to evaluate MS/MS clustering performance and DreaMS attention heads, can be downloaded from the official Hugging Face repository (https://huggingface.co/datasets/roman-bushuiev/MassSpecGym).

Research involving human participants, their data, or biological material

Policy information about studies with human participants or human data. See also policy information about sex, gender (identity/presentation), and sexual orientation and race, ethnicity and racism.

Reporting on sex and gender	N/A
Reporting on race, ethnicity, or other socially relevant groupings	N/A
Population characteristics	N/A
Recruitment	N/A
Ethics oversight	N/A

Note that full information on the approval of the study protocol must also be provided in the manuscript.

Field-specific reporting

Please select the one below that is the best fit for your research. If you are not sure, read the appropriate sections before making your selection.

☒ Life sciences ☐ Behavioural & social sciences ☐ Ecological, evolutionary & environmental sciences

For a reference copy of the document with all sections, see nature.com/documents/nr-reporting-summary-flat.pdf

Life sciences study design

All studies must disclose on these points even when the disclosure is negative.

Sample size	The sample size for our machine learning model was determined by the availability of MS/MS spectra from the public MassIVE GNPS repository, which contains tens to hundreds of millions of spectra, as well as from curated datasets MoNA and NIST20, which include approximately one million mass spectra in
Data exclusions	We excluded mass spectra acquired in negative ionization mode and those corresponding to multiply charged ions. This decision was made because our work focuses solely on small-molecule mass spectrometry in positive ion
Replication	To ensure the reproducibility of our method, we have provided detailed tutorials outlining how to replicate each step using our codebase, available at: https://dreams-docs.readthedocs.io.
Randomization	To train our machine learning model, we employed a standard mini-batch gradient descent technique, which involves random sampling of training examples from the entire dataset. The random seeds used in our experiments are fixed in our codebase.
Blinding	Blinding was not applicable in this study as it involved training and testing a machine learning model on pre-existing datasets, without any human intervention or subjective bias in the data allocation process.

Reporting for specific materials, systems and methods

We require information from authors about some types of materials, experimental systems and methods used in many studies. Here, indicate whether each material, system or method listed is relevant to your study. If you are not sure if a list item applies to your research, read the appropriate section before selecting a response.

Materials & experimental systems

n/a	Involved in the study
☒	☐ Antibodies
☒	☐ Eukaryotic cell lines
☒	☐ Palaeontology and archaeology
☒	☐ Animals and other organisms
☒	☐ Clinical data
☒	☐ Dual use research of concern
☒	☐ Plants

Methods

n/a	Involved in the study
☒	☐ ChIP-seq
☒	☐ Flow cytometry
☒	☐ MRI-based neuroimaging

Plants

Seed stocks	N/A
Novel plant genotypes	N/A
Authentication	N/A
